# Intron exon boundary junctions in human genome have in-built unique structural and energetic signals

**DOI:** 10.1093/nar/gkab098

**Published:** 2021-02-23

**Authors:** Akhilesh Mishra, Priyanka Siwach, Pallavi Misra, Simran Dhiman, Ashutosh Kumar Pandey, Parul Srivastava, B Jayaram

**Affiliations:** Supercomputing Facility for Bioinformatics & Computational Biology, Indian Institute of Technology Delhi, India; Kusuma School of Biological Sciences, Indian Institute of Technology, Delhi, India; Supercomputing Facility for Bioinformatics & Computational Biology, Indian Institute of Technology Delhi, India; Department of Biotechnology, Chaudhary Devi Lal University, Sirsa, Haryana, India; Supercomputing Facility for Bioinformatics & Computational Biology, Indian Institute of Technology Delhi, India; Supercomputing Facility for Bioinformatics & Computational Biology, Indian Institute of Technology Delhi, India; Kusuma School of Biological Sciences, Indian Institute of Technology, Delhi, India; Supercomputing Facility for Bioinformatics & Computational Biology, Indian Institute of Technology Delhi, India; Supercomputing Facility for Bioinformatics & Computational Biology, Indian Institute of Technology Delhi, India; Kusuma School of Biological Sciences, Indian Institute of Technology, Delhi, India; Department of Chemistry, Indian Institute of Technology, Delhi, India

## Abstract

Precise identification of correct exon–intron boundaries is a prerequisite to analyze the location and structure of genes. The existing framework for genomic signals, delineating exon and introns in a genomic segment, seems insufficient, predominantly due to poor sequence consensus as well as limitations of training on available experimental data sets. We present here a novel concept for characterizing exon–intron boundaries in genomic segments on the basis of structural and energetic properties. We analyzed boundary junctions on both sides of all the exons (3 28 368) of protein coding genes from human genome (GENCODE database) using 28 structural and three energy parameters. Study of sequence conservation at these sites shows very poor consensus. It is observed that DNA adopts a unique structural and energy state at the boundary junctions. Also, signals are somewhat different for housekeeping and tissue specific genes. Clustering of 31 parameters into four derived vectors gives some additional insights into the physical mechanisms involved in this biological process. Sites of structural and energy signals correlate well to the positions playing important roles in pre-mRNA splicing.

## INTRODUCTION

Discovery of eukaryotic genes as discontinuous structures, with protein-coding segments or exons disrupted by non-protein coding segments or introns, was one of the most unanticipated findings in molecular biology (1), whose mystery is yet to be solved fully. In fact, identification of genes with correct exon–intron architecture is one of the hardest problems in eukaryotic genome annotation. The key signals used for this purpose are the splice-site (SS) sensors which conventionally include a G–T sequence signal at the 5′SS (+1/+2 position at the 5′ end of intron) and A–G sequence signal at the 3′SS (last two positions at the 3′ end of intron). There exists a plethora of sequence variations at these sites, considering that thousands of different sequences act as naturally occurring splice sites in the human transcriptome (2), along with many variably located cryptic SSs (3). This greatly reduces the accuracy and fidelity of SS sequence signals for the identification of precise exon–intron boundaries, and the situation becomes more challenging in the wake of alternative splicing happening so prevalently in eukaryotes (4). The exact locations of the exon–intron boundaries are crucial not only for defining the encoded amino acid sequence but also for understanding the molecular mechanism underlying the regulation of pre-mRNA splicing, which is fundamental to understand gene expression and is of great medical relevance as at least 15% of human genetic disorders and many diseases are caused by aberrant pre-mRNA splicing (5).

Over the years, computational methods have emerged as a major force in fast and accurate characterization of genes/genomic segments. Some algorithms have been developed which determine a splice site based on a score calculated by measuring its concordance to matrices built using large collections of splice sites (6–9). Earlier gene prediction tools like Genscan, Genomescan combine exon–intron and splice signal models with similarity to known protein sequences in an integrated mode for gene predictions (10–12). Some other important tools like Genewise, Genomewise (13), Augustus (14), Fgenesh (15), GeneParser (16), GeneID (17) are *ab initio* gene prediction tools which have been developed using various programming models (Dynamic or Hidden Markov Model) on sequence information for functional signals including splice sites. These methods have been developed on huge training data and performance is high for a species/organism but for a naïve genome/genomic fragment, it decreases considerably. Some currently available splice-junction prediction tools identify exon–intron boundaries in mRNA sequences, for organisms with reference genome (18–21) as well as without a reference genome (22), but these tools are unable to annotate splice junctions in DNA sequence. Recently, role of chromatin organization and nucleosome positioning as determinant of exon–intron boundary was also investigated; though it looks at the problem with a new angle, satisfactory level of sensitivity and specificity could not be achieved (23). Despite the many insights resulting from such studies over the years, it is apparent that our conceptual frameworks are not adequate yet. New ideas and models are needed for identification of splice sites in genome sequences.

So, if the discriminatory signals for exon–intron boundaries are not uniformly present in the corresponding sequence, where to look for such signals? It is a well-known fact that DNA in living cell is not a uniform linear macromolecule but displays local structural and energetic variations which have been found to facilitate interactions with proteins and play a key role in several biological processes (24). The known structural biology of B-form DNA advanced dramatically with the solution of the crystal structure of the B-form oligonucleotide duplex *d*(CGCGAATTCGCG) in 1981, indicating the first observation of sequence dependent structural heterogeneity at the molecular level (25). Considerable subsequent efforts to gather data pertaining to sequence effects on the structure, during the last few decades, have led to revolutionary evolution in the analysis of nucleic acids structure (26–33). Many studies over the years have found that similar sequences may lead to similar structure and energetics, but reverse is not true however, different sequences can lead to DNA molecules with similar structure and energetic properties (34–35). Do exon–intron boundaries too represent a similar case where DNA attains a uniform and unique structural and energetic state, despite the presence of huge sequence variations, at these sites? But why would DNA structure and energetics change at exon–intron boundaries as splicing is an affair between pre-mRNA and spliceosome (a dynamic macromolecular machine composed of five small nuclear RNAs, associated polypeptides and many other protein factors) and there is never a direct interaction between spliceosome and DNA and so, this idea initially seemed unlikely. However, while carrying out literature survey, we started getting clues. Some studies have shown that exons have higher thermodynamic stability compared to introns, untranslated regions (UTRs) and intergenic regions, (36–37). Though these studies do not investigate the energetics of splice sites, they indicate that exon–intron boundaries might show some signal depicting the transition in thermodynamic property from exon to intron or vice versa. Further, a large number of evidences have shown that pre-mRNA splicing is pre-dominantly co-transcriptional (38–41). Evidences exist for kinetic coupling (42–44) as well as physical and mechanistic coupling (45–46) between transcription and splicing. These studies are indicating an indirect link between DNA template and splicing. Do structure and energetics of DNA template at exon–intron boundaries offer some mechanisms to regulate both–the elongation rate of pre-mRNA as well as splicing of upstream intron, or, are they offering some platform to physically/mechanistically link the RNA polymerase II and spliceosome? Before going any further in this direction, it became imperative that the structural and energetic behavior of exon–intron boundaries be investigated. Since these signals do not manifest directly in the sequence itself, previous studies pertaining to sequence analysis of exon–intron boundaries (6–17) do not offer information regarding the structure and energy signals of boundary junctions.

Over the years, some very remarkable methods have become available for the analysis of nucleic acids structure (26–33). During the last 15 years, we have also made some significant efforts to understand the DNA language in terms of its energetics and structure (47–55). For the present study, we proceeded by downloading all the exons (3 28 368) from protein coding genes of human genome from GENCODE database and obtained the genomic coordinates of exon-start and exon-end position. Using these genomic coordinates, two boundary sequences datasets were prepared- Dataset I and Dataset II, each having 3 28 368 sequences of length 401 nucleotides (detail in method section). These boundary sequences were subjected to structural and energetic characterization using 28 structural and three energy parameters. To obtain numeric values of conformational parameters for the unique di-nucleotides steps, we downloaded the crystals structures of B-DNA from Nucleic Acids Database (NDB) (55) and applied the Curves+ webserver (31) on these structures for the same. In-house programs were used for calculating the energy parameters (53). Here, we report that these parameters provide unique structural and energetic signatures at SS junctions and the information for these signatures is in-built in their sequences. Our results offer a whole new paradigm for understanding pre-mRNA splicing which can go a long way in understanding regulation of eukaryotic gene expression.

## MATERIALS AND METHODS

### Boundary sequence dataset

Genome annotation file of human genome was downloaded from GENCODE database and from this, all the exons (3 28 368) from protein coding genes were extracted, and for each exon–exon-start and exon-end genomic coordinates were taken out. Using these genomic coordinates, two datasets for boundary sequences were prepared: Dataset I and Dataset II. Dataset I was prepared by extracting 401 nucleotides, spanning 200 nucleotides upstream and downstream, with respect to the exon end position, taking it as ‘0’; these sequences, each of length 401 nucleotides, represent exon sequence from −200 to 0 and intron sequence from +1 to +200. Likewise, Dataset II was prepared with respect to exon start position (the sequences here represent intron sequence from −200 to −1 and exon sequences from 0 to +200). In this way, each dataset has 3 28 368 sequences, of length 401 nucleotides each. As control dataset (Dataset III), we extracted 30 140 sequences of length 401 nucleotides from the middle of exons, which are >1000 nucleotides long (www.scfbio-iitd.res.in/chemgenome/intron_exon).

### Parameters for characterization of genomic sequences

We have used 28 structural and three energetic parameters. The structural parameters include—nine backbone (Alpha, Beta, Gamma, Delta, Epsilon, Zeta, Chi, Phase and Amplitude), eight inter-BP (Shift, Slide, Rise, Tilt, Roll, Twist, H-Rise and H-Twist), six intra-BP (Shear, Stretch, Stagger, Buckle, Propel and Opening) and five BP-axis (X Displacement, Y Displacement, Inclination, Tip and Axis-Bend) parameters. The values of these parameters were calculated by applying Curves+ webserver (44) on 74 B-DNA crystal structures obtained from NDB database (Supplementary Table S1.1) (55). After calculating values for all the parameters for each B-DNA structure, all occurrences of unique 10 di-nucleotide steps in the 5′ to 3′ direction were considered for each parameter and the average of all the occurrences were calculated. Proper methods were used for the statistical analysis of angular values (56–58).

The energy parameters include- hydrogen bond energy, stacking energy and solvation energy. The values for these three energy parameters for the unique 10 di-nucleotide steps was done as reported in our previous work (53).

The numeric values, of all the 30 parameters, for the unique di-nucleotides steps, obtained above are provided in supplementary Table S1.2. All the numeric conversions of the present study were made according to this table.

### Obtaining the structural and energy numeric profile of each sequence

The calculated di-nucleotide values for each parameter (from Supplementary Table S1.2) were used for getting numeric profile of each sequence of all the three datasets by performing moving average calculation on a sliding window of 25 bp covering 24 di-nucleotide steps (the first element of the moving average is obtained by taking the average of the initial first 24 di-nucleotide steps then the window is shifted forward, excluding the first number and including the next set of 24 di-nucleotide steps) (selection of the 25-bp window size was based on initial screening of sample data with window sizes of 15, 20, 25 and 30). The same exercise was performed independently on all the selected sequences for all the 31 parameters. In this way, 31 numeric profiles were obtained for each of the 3 28 368 sequences, for both the datasets: Dataset I and Dataset II. Likewise, all sequences of Dataset III (CDSs) were also subjected to numeric profile generation; 31 numeric profiles were generated for each sequence. [The term ‘Profile’ here is used for the unique set of numeric values for each nucleotide position (from −200 to +200, through 0) along the length of the sequence.] Data is available in raw csv format as supplementary file 2.

### Normalization of values

To bring all the parameters on the same scale, the values were made dimensionless using normalization. The values were normalized between 0 and 1 by subtracting the minimum value of the profile from each value and then by dividing the value with the range of the profile (i.e. max – min).

### Error analysis of data

The standard error of the mean at each position from −200 to +200 for all the parameters was calculated by dividing standard deviation of values at that position divided by square root of total number of observations. The standard error along with mean value is presented in Supplementary Figure S1.1a–e and S1.2a–e as shaded error bars.

### Profile plotting of sequences

The plotting was performed using MATLAB software.

### Examining the observations on individual sequences

To examine the generality of observations on individual sequences of Datasets I and II, following methodology was used.

For sequences of both datasets, for each parameter, a vector of 61 residues in length (spanning −30 to +30 through 0) was taken and was named as junction vector. To generate the CDS vector (as control, for comparison), for every position in the junction vector, a relative position towards the exon region was mapped at 150 residues away from it. For Dataset I (exon from −200 to 0 and intron from +1 to +200) the control vector was upstream of junction vector while for Dataset II (intron from −200 to +1 and exon from 0 to +200) control vector was downstream of junction vector. Then for every pair of junction vector and corresponding CDS vectors, the area enclosed by them was calculated. Logic is that, those pairs of junctions and CDS vectors where area enclosed by them is small (<2 standard deviations from the mean) will be indistinguishable, whereas vice versa is true for pairs having area greater than this value. Thus, sequences which qualified the threshold criteria of (mean – 2 × standard deviations) for the area calculated, were selected as having significant junction signals and those not meeting the threshold criteria were considered as sequences not having the signal. Formulas for calculation of area under the curve and optimization process of threshold values are given in supplementary methodology S1.1a, b and Supplementary Figures S1.3–S1.4.

### Signals in housekeeping genes and tissue specific genes

In order to compare the signals, at splice junctions, of housekeeping genes with those of tissue specific genes, we obtained the complete list of 53 exons from 11 housekeeping genes (59) and 141 exons from 11 tissue specific genes (top 6 brain specific and top 5 liver specific genes) (60). With respect to the exon start and end position in each case, 200 nucleotides were extracted from each side from the corresponding genomic sequence, as explained earlier, to prepare two datasets for both housekeeping genes (Dataset_HK I and Dataset_HK II) and tissue specific genes (Dataset_TS I and Dataset_TS II). Plotting was done as explained earlier.

### Clustering the data into sets of few plots

To simplify the data to facilitate a better interpretation, the data of 31 plots for both the Datasets were clustered into a set of a few plots. Data were sorted out by the value near position −25, identified as having positive or negative values (and slopes). Five parameters (Y-displacement, Opening, Delta, stacking energy and Solvation energy) did not match these profiles and so were eliminated. Rest 26 parameters were clustered in two groups, based on the value near −25 position. Group I represents those parameters which exhibited an increase near −25 position and included 13 parameters-Stretch, Rise, Tilt, Roll, Twist, H-rise, H-twist, beta, Gamma, Epsilon, Phase, Amplitude, Hydrogen Bond Energy. While 13 parameters (X-displacement, Inclination, Tip, ax-Bend, Shear, Stagger, Buckle, Propeller twist, Shift, slide, alpha, Zeta and Chi) showed a decrease in value near −25 position and were clustered together as group II. The plots of these two groups for both the Datasets-I and II, were generated by scaling each data set as follows: (data – average(data)/(max(data) – min(data)).

## RESULTS AND DISCUSSIONS

**Figure 1.**
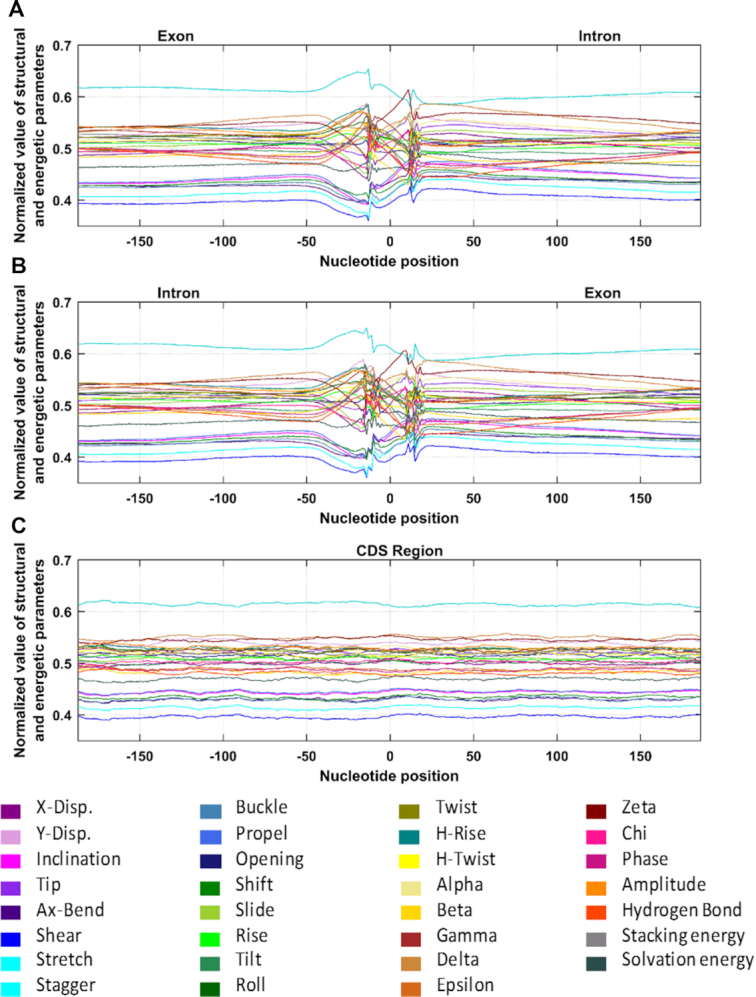
Average profiles for 31 parameters, obtained by plotting the normalized values, for (**A**) Dataset I (3 28 368 sequences, showing transition from exon to intron as first half of the sequence, i.e. −200 to 0 is exon sequence while second half, i.e. from +1 to +200 is intron sequence). (**B**) Dataset II (3 28 368 sequences showing transition from intron to exon) and (**C**) Dataset III (30 140 coding sequences). The average profile for a given parameter was obtained by superimposing numeric profiles of all the individual sequences belonging to a particular dataset and calculating the average over all numeric sequences for each position. The ordinate represents the normalized values while the abscissa shows the nucleotide position.

Numeric profiles of 31 parameters (28 structural and 3 energy) were obtained for pooled sequences of each dataset: Dataset I (3 28 368 sequences), Dataset II (3 28 368 sequences) and Dataset III (30 140 sequences) (each sequence of length 401 nucleotides). For this, for each parameter, numeric profiles of all the individual sequences belonging to a particular dataset were superimposed and average over all numeric sequences for each position was calculated. In this way, for each dataset, we obtained 31 average numeric profiles and these average profiles were then used for the plotting purpose, with abscissa showing nucleotide position and ordinate representing the numeric value of that parameter (Supplementary Figures S1.1–S1.2a–e, parameter-wise plot for the three datasets (Datasets I, II and III), showing the error bars too). To evaluate all the parameters on single scale, values were normalized and all the 31 normalized parameters were plotted together on this new scale, for the three datasets-Datasets I, II and III. (Figure 1).

It is clear from Figure 1 that for all the 31 parameters, a unique pattern is observed from −50th to +25th position, which is quite distinct from the corresponding upstream and downstream regions, indicating a considerable change in DNA structural and energetic properties at these locations. Figure 1a represents the signal obtained for Dataset I (exon sequence from −200 to 0 and intron sequence from +1 to +200), showing the parameter profile as we move from exon to intron while Figure 1B represents the profile of Dataset II (intron sequence from −200 to −1 and exon sequence from 0 to +200) as sequence transitions from intron to exon. It is quite notable that though change in values of each parameter starts happening from around −50th position, the pattern of change is quite unique for each parameter for both the datasets; for some parameters, values increase initially followed by sudden decrease and the reverse for others. On the other hand, the plots of CDSs (Dataset III), as shown in Figure 1C, come as straight lines, with no changes occurring anywhere across the entire length of sequence (parameter wise value is given in Supplementary File S2). This clearly suggests that DNA undergoes a distinct change in its structure and energy as it transitions from exon to intron and vice versa while no such change occurs across the length of CDS.

**Figure 2.**
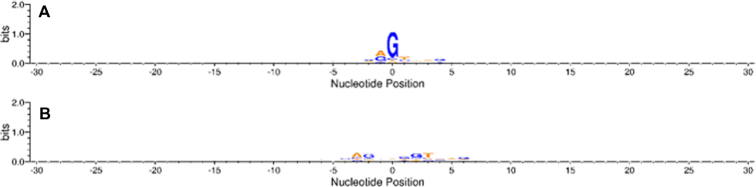
Sequence consensus, as observed using WebLogo software, at positions ‘−30 to +30’ of 3 28 368 sequences, within each dataset, (**A**) Dataset I and (**B**) Dataset II.

Since Figure 1 is an average plot of all the sequences of a particular dataset, it becomes imperative to know the generality of this observations across the individual sequences. Using the methodology as explained in method section, it was observed that for both the datasets, the signal for each parameter, at positions ‘−30 to +30’ was observed in >95% of the sequences (detailed results are available in Supplementary Table S1.3 and S1.4). A distribution plot of area calculated for every pair of junction vector and CDS vector (3 28 368) is not feasible, with such a large data. The observation of structural and energy signals on such a large percentage of data led us to investigate the situation at sequence level too. We wanted to know whether at these positions (i.e. −30 to +30) some consensus exists at sequence level or not. For this, sequences within each dataset (Dataset I and Dataset II) were aligned from −30 to +30 positions and consensus was observed using WebLogo3 software (61) (Figure 2).

It is very clear from Figure 2 that for both the datasets, some consensus is observed only at positions ‘from −5/4 to +6/7’, whereas for rest of the positions, there is no consensus. The results are clearly indicating towards the universality of structure and energy signals compared to sequence signals for splice site identification. The presently available methods (6–17) for splice site predictions are predominantly based on sequence information for various functional regions at and near to the acceptor and donor splice site. However, since the present study does not offer the designing of a method/algorithm for splice site prediction (though it is the eventual target of our study), rather represents the first stage only (dealing with the identification and characterization of structure and energy signals at splice sites), a comparison with the existing methods for prediction is not possible at this stage.

**Figure 3.**
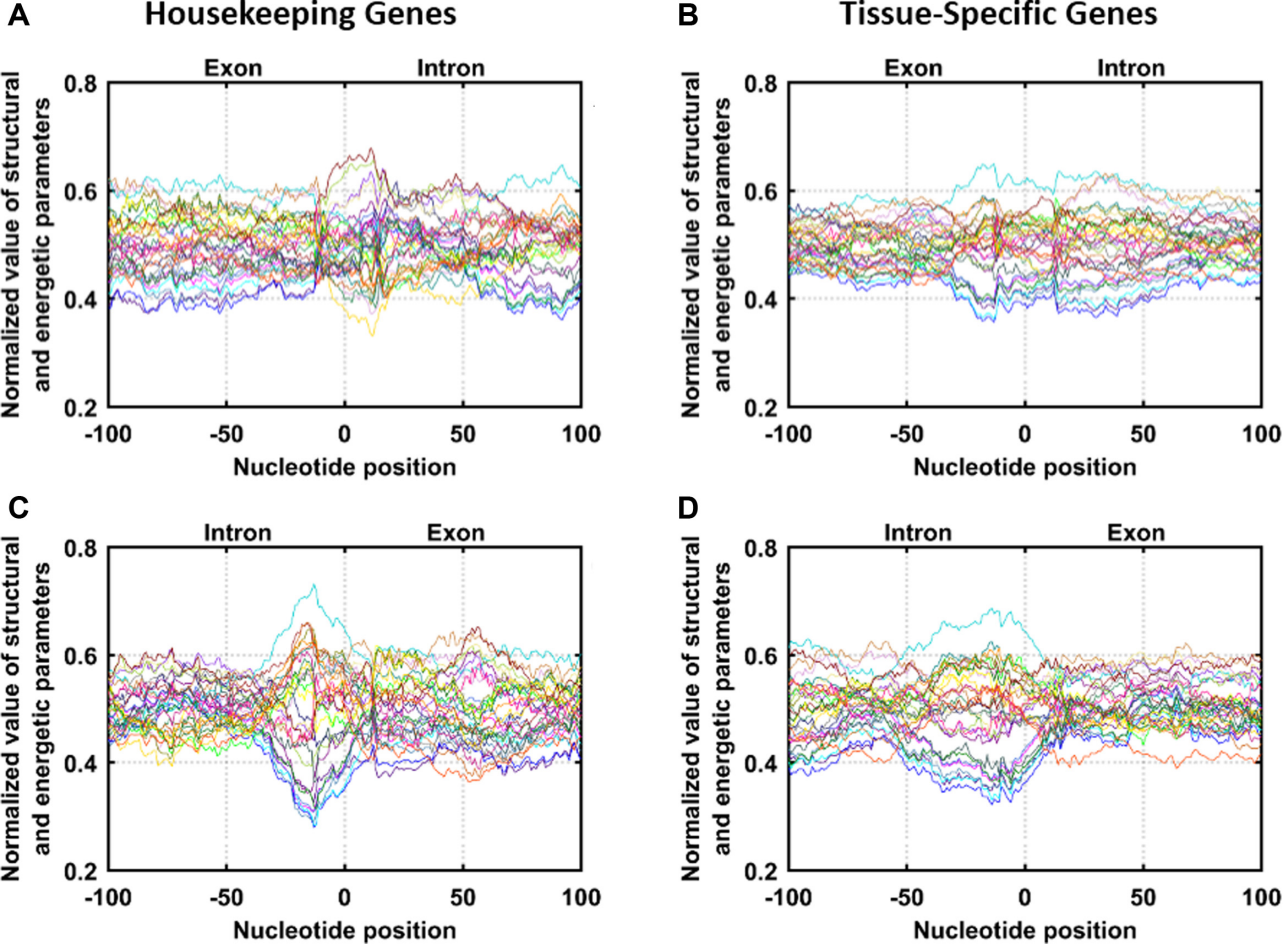
Average profiles obtained for sequences of housekeeping (HK) and tissue specific (TS) genes: (**A**) Dataset_HK_I (53 sequences of length 201 nucleotides, exon sequence from −100 to 0 and intron from +1 to +100), (**B**) Dataset_TS_I (141 sequences of 201 nucleotides length, exon sequence from −100 to 0 and intron from +1 to +100), (**C**) Dataset_HK II (53 sequences of length 201 nucleotides, intron sequence from −100 to −1 and exon sequence from 0 to +100), (**D**) Dataset_TS II (141 sequences length 201 nucleotides, intron sequence from −100 to −1 and exon sequence from 0 to +100).

When signals at splice junctions were compared for housekeeping versus tissue specific genes, comparatively sharper and distinct signals were observed for housekeeping genes (Figure 3A and C) than tissue specific genes (Figure 3B and D) (Figure 3).

Further, for both types of genes, signals were sharper when sequences move from intron to exon (Figure 3C and D) compared to exon to intron (Figure 3A and B). This observation can lead to some deeper insights into the role of in-built designs of genes in gene expression, though it is difficult to comment further on this issue with the present set of observations. Further studies are needed to give clear insights on this aspect.

We attempted to understand our findings in the light of existing mechanisms for pre-mRNA splicing. Two unique spliceosomes coexist in most eukaryotes. We preferred the most common mechanism involving U2-dependent spliceosome, ignoring the one with less abundant U12-dependent spliceosome which is present in only a subset of eukaryotes (62).

**Figure 4.**
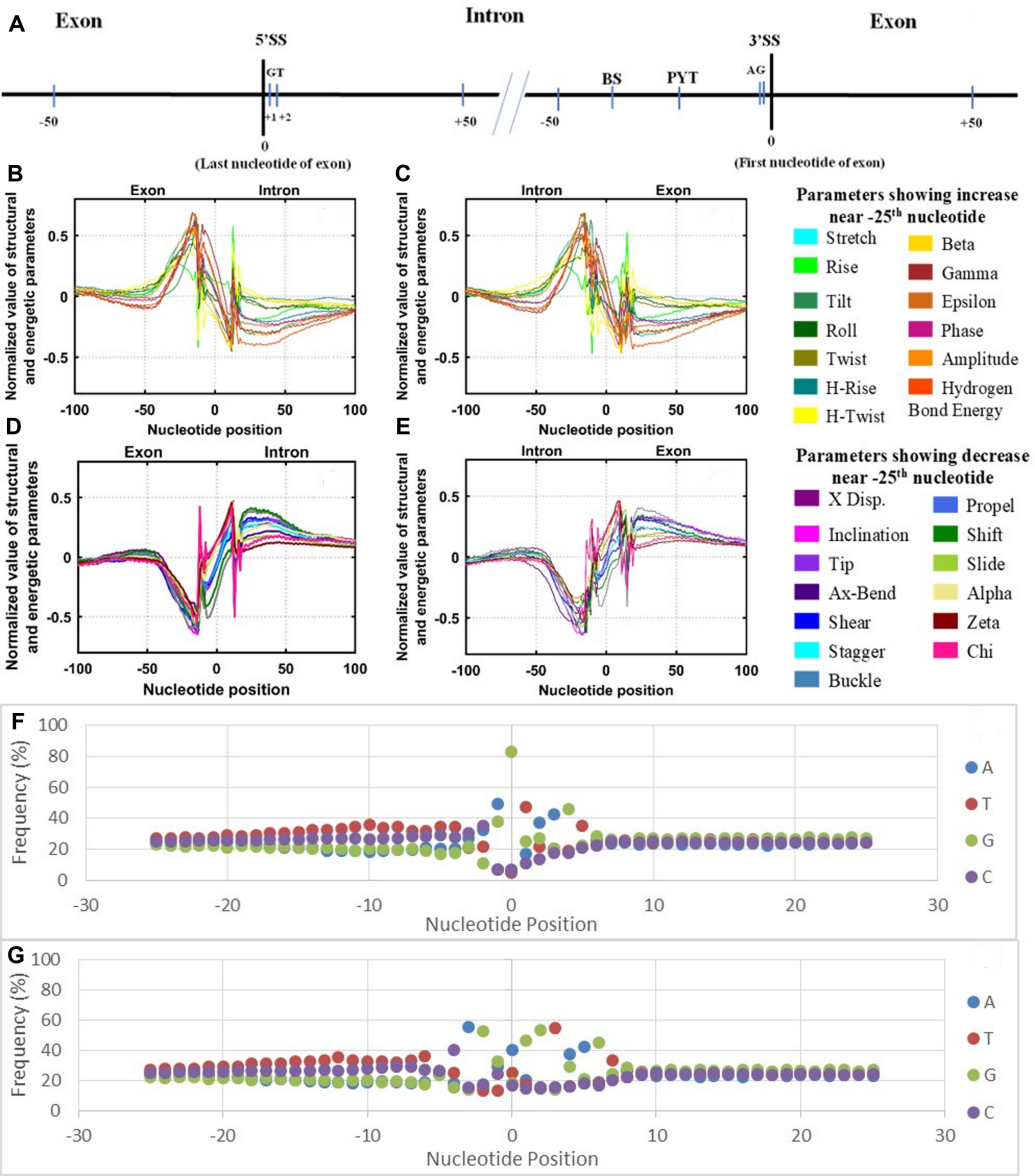
Pictorial representation of a genomic fragment (5′ to 3′) showing positions of two splice sites: 5′SS (from exon to intron, first two positions of intron are generally occupied by GT), and 3′ SS (from intron to exon, with last two positions of intron generally having AG sequence). The last 50 nucleotides of intron have two important cis-acting elements: Branch-point (BS), generally located 18–40 nucleotide upstream of 3′SS and polypyrimidine tract (PYT) between BS and 3′SS (**A**). Normalized values of 13 parameters showing increase near −25 positions in Dataset I (**B**) and Dataset II (**C**). Normalized values of 13 parameters showing decrease near −25 position in Dataset I (**D**) and Dataset II (**E**). Position-wise frequency of four bases for a region of −25 to +25 with respect to each splice site for both datasets: Dataset I (**F**) and Dataset II (**G**).

There are many *cis*-acting elements present on both sides of splice junctions which play important role in spliceosome assembly and splicing (Figure 4A). These include branch site (BS), polypyridine tract (PYT), exonic and intronic splicing enhancers (ESEs and ISEs) or silencers (ESSs and ISSs). The BS is typically located 18–40 nucleotides upstream from the 3′SS while PYT is present variably between BS and 3′SS. Location and sequence of silencers and enhancers is highly variable.

To correlate these important sequence positions to the corresponding state of DNA structure and energy, we put a simplified structure and energy parameter plot (involving 26 parameters only) just below it (as Figure 4B–E) (details in method section). Further, to gain some insights into the underlying correlations/anti-correlations among these 26 parameters, position specific correlation coefficients were calculated for the positions from −25 to −35, for both the datasets (Supplementary File S3) and corresponding heat plots were also generated (Supplementary Figure S1.5). For Dataset I, all the pairs exhibited strong correlations/anti-correlations; correlation coefficients values ranged from 0.911 (between Tilt and Rise) to 1 (for the pairs of Buckle-Inclination, Propel-Inclination, Propel-Buckle, Shift-Stagger, Slide-X-Disp., Delta-Gamma, Phase-Delta, Amplitude-H-bond energy, X-Disp-Inclination, Tip-Ax-bend) while anti-correlation coefficient values were observed in the range of −0.942 (for the pairs of Tilt-Tip, Tilt-Ax-bend) to −1 (for the pairs of Stretch-Shear, Stagger-Stretch, Zeta-Gamma, Zeta-Delta, Phase-Zeta, H-bond energy-Inclination, H-bond energy-Propel). In Dataset II, moderate to high level of correlations/anti-correlations were observed between various pairs of parameters, as evident from Supplementary file S3; correlation coefficients values ranged from 0.528 (between Rise and Tilt) to 1 (for the pairs of Propel-Inclination, Beta-Inclination, Propel-Stagger, Beta-Propel, Zeta-Slide, Gamma-Delta, Gamma-Phase, Delta-Phase) whereas anti-correlation coefficient values varied from −0.634 (between Rise-Slide) to −1 (for the pairs of Gamma-X-Disp., Amplitude-Shift, Slide-Gamma, Slide-Epsilon, Beta-H-rise, Gamma-Zeta, Zeta-Phase). Such an observation opens up gates for understanding many underlying mechanisms governing DNA structure as well as the purpose behind the changes in various structural parameters at the intron-exon boundary junctions, definitely calling for more research for further clarity. Some changes corroborated well with the established facts like—the negative coupling of two pairs of dihedral angles: epsilon-zeta (epsilon increased while zeta decreased) (63) and alpha–gamma (alpha decreased while gamma increased (64), which are associated with change of DNA structure from canonical to non-canonical state.

Spliceosome assembly occurs by the ordered interaction of the spliceosomal snRNPs (small nuclear ribonucleic proteins) and numerous other splicing factors (SF) (65–66). In the first step, the 5′SS is recognized by U1 snRNP, the BS by SF1, and the PYT by U2AF65 (SF). The BS and PYT show poor sequence consensus while for the −3 to +6 region of the 5′ SS, >9000 sequence variants have been recently recorded (67). Our effort to find the nucleotide frequency at individual positions, from −25 to +25 with respect to each splice site, also revealed no significant consensus frequencies for these positions (Figure 4F and G) (Position wise frequency data is given in Supplementary File S4). What then drives the initial identification of these three important sites? Figure 4B–E indicate towards a unique structural and energetic state of DNA at these positions which might act as the identification signal for these sites. In recent times, many studies have emerged which show that DNA structural and energetic properties greatly aid the targeting and functionality of DNA-binding proteins in a wide variety of ways (24,68).

In the second step of spliceosome assembly, the U2 snRNP joins BS by replacing SF1 (forms the A complex), followed by subsequent joining of the U4/U6.U5 tri-snRNP (the B complex); extensive structural rearrangements occur at this stage to activate the spliceosome. After the rearrangements (where U1 and U4 snRNP leave the assembly), the U6 snRNP directly interacts with 5′SS and U5 snRNP directly interacts with 3′SS. Since U6 and U5 are linked, being part of tri-snRNPs, it brings 5′SS and 3′SS in a juxtaposed orientation (activated B complex). It is followed by first trans-esterification reaction at 5′SS, resulting in formation of C complex where second catalytic reaction occurs at 3′SS, resulting in release of intron and ligation of exon ends. In the light of the results obtained in the present study, it can be speculated that exact pattern of structural and energy changes occurring at both ends of intron (Figures 1 and 4) might have some role to bring the two ends of intron in juxtaposed orientation; more studies are definitely needed to corroborate the fact.

The above results, undoubtedly, affirm the active role of DNA structure and energetics at the SS junctions, though from the present study it is difficult to interpret the exact nature of their role. On further contemplation, a surge of queries emerges. What type of structure and energy state DNA adopts at SS junctions? Why is the DNA undergoing such changes at SS junctions when it is not directly interacting with spliceosome? How RNA polymerase II responds to this change in template? Does splicing of pre-mRNA at SSs is affected/stimulated by structure and energy states of corresponding sites at template DNA, if yes then how? Numerous evidences do exist for post-transcriptional mechanisms of pre-mRNA splicing, but the role of DNA template-based changes is unclear. Attempts to answer above questions would reveal fundamentally new insights into the regulation of gene expression. We plan to address some of these questions in near future and anticipate that many new dimensions would be added by the scientific community involved in similar work.

## CONCLUSION

Structural and energy analysis of 6 56 736 genomic sequences, pertaining to exon–intron boundary sites (3 28 368 sequences of each type—exon to intron and vice versa) clearly points to the existence of physico-chemical fingerprints for these locations, irrespective of whether consensus exists at sequence level or not. Identification of precise splice sites in eukaryotic genes/genome annotation has remained a big challenge till date because of poor sequence consensus at these sites. Using the observations of present study, we hope to develop an efficient algorithm/method for splice site prediction. Existence of physico-chemical fingerprints conveying the functional destiny of DNA sequences has earlier been used for identification of many elements, like promoters, operator/regulators as well as for different classes of RNAs, in genome/genomic segments by many scientific groups.

## Supplementary Material

gkab098_Supplemental_Files

## References

[1] Sharp P.A. Split genes and RNA splicing. Cell. 1994; 77:805–815. 7516265 10.1016/0092-8674(94)90130-9

[2] Roca X. , Krainer A.R. Recognition of atypical 5′ splice sites by shifted base-pairing to U1 snRNA. Nat. Struct. Mol. Biol. 2009; 16:176–182. 19169258 10.1038/nsmb.1546 PMC2719486

[3] Roca X. , Sachidanandam R., Krainer A.R. Intrinsic differences between authentic and cryptic 5′ splice sites. Nucleic Acids Res. 2003; 31:6321–6333. 14576320 10.1093/nar/gkg830 PMC275472

[4] Liu Y. , Gonzàlez-Porta M., Santos S., Brazma A., Marioni J.C., Aebersold R., Venkitaraman A.R., Wickramasinghe V.O. Impact of alternative splicing on the human proteome. Cell Rep. 2017; 20:1229–1241. 28768205 10.1016/j.celrep.2017.07.025 PMC5554779

[5] Anna A. , Monika G. Splicing mutations in human genetic disorders: examples, detection, and confirmation. J. Appl. Genet. 2018; 59:253–268. 29680930 10.1007/s13353-018-0444-7 PMC6060985

[6] Senapathy P. , Shapiro M.B., Harris N.L. Splice junctions, branch point sites, and exons: sequence statistics, identification, and applications to genome project. Methods Enzymol. 1990; 183:252–278. 2314278 10.1016/0076-6879(90)83018-5

[7] Brunak S. , Engelbrecht J., Knudsen S. Prediction of human mRNA donor and acceptor sites from the DNA sequence. J. Mol. Biol. 1991; 220:49–65. 2067018 10.1016/0022-2836(91)90380-o

[8] Yeo G. , Burge C.B. Maximum entropy modeling of short sequence motifs with applications to RNA splicing signals. J. Comput. Biol. 2004; 11:377–394. 15285897 10.1089/1066527041410418

[9] Sahashi K. , Masuda A., Matsuura T., Shinmi J., Zhang Z., Takeshima Y., Matsuo M., Sobue G., Ohno K. In vitro and in silico analysis reveals an efficient algorithm to predict the splicing consequences of mutations at the 5′ splice sites. Nucleic Acids Res. 2007; 35:5995–6003. 17726045 10.1093/nar/gkm647 PMC2094079

[10] Burge C. , Karlin S. Prediction of complete gene structures in human genomic DNA. J. Mol. Biol. 1997; 268:78–94. 9149143 10.1006/jmbi.1997.0951

[11] Burge C.B. Chapter 8 - Modeling dependencies in pre-mRNA splicing signals. New Compr. Biochem. 1998; 32:129–164.

[12] Yeh R.F. , Lim L.P., Burge C.B. Computational inference of homologous gene structures in the human genome. Genome Res. 2001; 11:803–816. 11337476 10.1101/gr.175701 PMC311055

[13] Birney E. , Clamp M., Durbin R. Genewise and genomewise. Genome Res. 2004; 14:988–995. 15123596 10.1101/gr.1865504 PMC479130

[14] Stanke M. , Morgenstern B. AUGUSTUS: a web server for gene prediction in eukaryotes that allows user-defined constraints. Nucleic Acids Res. 2005; 33:W465–W467. 15980513 10.1093/nar/gki458 PMC1160219

[15] Salamov A.A. , Solovyev V.V. Ab initio gene finding in Drosophila genomic DNA. Genome Res. 2000; 10:516–522. 10779491 10.1101/gr.10.4.516 PMC310882

[16] Snyder E.E. , Stormo G.D. Identification of coding regions in genomic DNA sequences: an application of dynamic programming and neural networks. Nucleic Acids Res. 1993; 21:607–613. 8441672 10.1093/nar/21.3.607 PMC309159

[17] Guigó R. , Knudsen S., Drake N., Smith T. Prediction of gene structure. J. Mol. Biol. 1992; 226:141–157. 1619647 10.1016/0022-2836(92)90130-c

[18] Trapnell C. , Pachter L., Salzberg S.L. TopHat: discovering splice junctions with RNA-Seq. Bioinformatics. 2009; 25:1105–1111. 19289445 10.1093/bioinformatics/btp120 PMC2672628

[19] Au K.F. , Jiang H., Lin L., Xing Y., Wong W.H. Detection of splice junctions from paired-end RNA-seq data by SpliceMap. Nucleic Acids Res. 2010; 38:4570–4578. 20371516 10.1093/nar/gkq211 PMC2919714

[20] Wang K. , Singh D., Zeng Z., Coleman S.J., Huang Y., Savich G.L., He X., Mieczkowski P., Grimm S.A., Perou C.M. et al. MapSplice: accurate mapping of RNA-seq reads for splice junction discovery. Nucleic Acids Res. 2010; 38:e178. 20802226 10.1093/nar/gkq622 PMC2952873

[21] Ameur A. , Wetterbom A., Feuk L., Gyllensten U. Global and unbiased detection of splice junctions from RNA-seq data. Genome Biol. 2010; 11:R34. 20236510 10.1186/gb-2010-11-3-r34 PMC2864574

[22] Levin L. , Bar-Yaacov D., Bouskila A., Chorev M., Carmel L., Mishmar D. LEMONS - a tool for the identification of splice junctions in transcriptomes of organisms lacking reference genomes. PLoS One. 2015; 10:e0143329. 26606265 10.1371/journal.pone.0143329 PMC4659627

[23] Fincher J.A. , Tyson G.S., Dennis J.H. DNA-Encoded chromatin structural intron boundary signals identify conserved genes with common function. Int. J. Genomics. 2015; 2015:167578. 25861617 10.1155/2015/167578 PMC4377520

[24] Rohs R. , West S.M., Sosinsky A., Liu P., Mann R.S., Honig B. The role of DNA shape in protein-DNA recognition. Nature. 2009; 461:1248–1253. 19865164 10.1038/nature08473 PMC2793086

[25] Dickerson R.E. , Drew H.R. Structure of a B-DNA dodecamer. II. Influence of base sequence on helix structure. J. Mol. Biol. 1981; 149:761–786. 6273591 10.1016/0022-2836(81)90357-0

[26] Yanagi K. , Prive G.G., Dickerson R.E. Analysis of local helix geometry in three B-DNA decamers and eight dodecamers. J. Mol. Biol. 1991; 217:201–214. 1988678 10.1016/0022-2836(91)90620-l

[27] el Hassan M.A. , Calladine C.R. The assessment of the geometry of dinucleotide steps in double-helical DNA; a new local calculation scheme. J. Mol. Biol. 1995; 251:648–664. 7666417 10.1006/jmbi.1995.0462

[28] Olson W.K. , Gorin A.A., Lu X.J., Hock L.M., Zhurkin V.B. DNA sequence-dependent deformability deduced from protein-DNA crystal complexes. PNAS. 1998; 95:11163–11168. 9736707 10.1073/pnas.95.19.11163 PMC21613

[29] Beveridge D.L. , Barreiro G., Byun K.S., Case D.A., Cheatham T.E. 3rd, Dixit S.B., Giudice E., Lankas F., Lavery R., Maddocks J.H. et al. Molecular dynamics simulations of the 136 unique tetranucleotide sequences of DNA oligonucleotides. I. Research design and results on d(CpG) steps. Biophys. J. 2004; 87:3799–3813. 15326025 10.1529/biophysj.104.045252 PMC1304892

[30] Dixit S.B. , Beveridge D.L., Case D.A., Cheatham T.E. 3rd, Giudice E., Lankas F., Lavery R., Maddocks J.H., Osman R., Sklenar H. et al. Molecular dynamics simulations of the 136 unique tetranucleotide sequences of DNA oligonucleotides. II: sequence context effects on the dynamical structures of the 10 unique dinucleotide steps. Biophys. J. 2005; 89:3721–3740. 16169978 10.1529/biophysj.105.067397 PMC1366942

[31] Lavery R. , Moakher M., Maddocks J.H., Petkeviciute D., Zakrzewska K. Conformational analysis of nucleic acids revisited: Curves+. Nucleic Acids Res. 2009; 37:5917–5929. 19625494 10.1093/nar/gkp608 PMC2761274

[32] Lavery R. , Zakrzewska K., Beveridge D., Bishop T.C., Case D.A., Cheatham T. 3rd, Dixit S., Jayaram B., Lankas F., Laughton C. et al. A systematic molecular dynamics study of nearest-neighbor effects on base pair and base pair step conformations and fluctuations in B-DNA. Nucleic Acids Res. 2010; 38:299–313. 19850719 10.1093/nar/gkp834 PMC2800215

[33] Pasi M. , Maddocks J.H., Beveridge D., Bishop T.C., Case D.A., Cheatham T. 3rd, Dans P.D., Jayaram B., Lankas F., Laughton C. et al. μABC: a systematic microsecond molecular dynamics study of tetranucleotide sequence effects in B-DNA. Nucleic Acids Res. 2014; 42:12272–12283. 25260586 10.1093/nar/gku855 PMC4231739

[34] Florquin K. , Saeys Y., Degroeve S., Rouzé P., Van de Peer Y. Large-scale structural analysis of the core promoter in mammalian and plant genomes. Nucleic Acids Res. 2005; 33:4255–4264. 16049029 10.1093/nar/gki737 PMC1181242

[35] Michael Gromiha M. , Siebers J.G., Selvaraj S., Kono H., Sarai A. Intermolecular and intramolecular readout mechanisms in protein-DNA recognition. J. Mol. Biol. 2004; 337:285–294. 15003447 10.1016/j.jmb.2004.01.033

[36] Kraeva R.I. et al. Stability of mRNA/DNA and DNA/DNA duplexes affects mRNA transcription. PLoS One. 2007; 2:e290. 17356699 10.1371/journal.pone.0000290 PMC1808433

[37] Nedelcheva-Veleva M.N. , Sarov M., Yanakiev I., Mihailovska E., Ivanov M.P., Panova G.C., Stoynov S.S. The thermodynamic patterns of eukaryotic genes suggest a mechanism for intron-exon recognition. Nat. Commun. 2013; 4:2101. 23817463 10.1038/ncomms3101

[38] Beyer A.L. , Osheim Y.N. Splice site selection, rate of splicing, and alternative splicing on nascent transcripts. Genes Dev. 1988; 2:754–765. 3138163 10.1101/gad.2.6.754

[39] Wuarin J. , Schibler U. Physical isolation of nascent RNA chains transcribed by RNA polymerase. II: evidence for cotranscriptional splicing. Mol. Cell. Biol. 1994; 14:7219–7225. 7523861 10.1128/mcb.14.11.7219-7225.1994 PMC359256

[40] Khodor Y.L. , Rodriguez J., Abruzzi K.C., Tang C.H., Marr M.T. 2nd, Rosbash M. Nascent-seq indicates widespread cotranscriptional pre-mRNA splicing in Drosophila. Genes Dev. 2011; 25:2502–2512. 22156210 10.1101/gad.178962.111 PMC3243060

[41] Pandya-Jones A. , Black D.L. Co-transcriptional splicing of constitutive and alternative exons. RNA. 2009; 15:1896–1908. 19656867 10.1261/rna.1714509 PMC2743041

[42] Fong N. , Kim H., Zhou Y., Ji X., Qiu J., Saldi T., Diener K., Jones K., Fu X.D., Bentley D.L. Pre-mRNA splicing is facilitated by an optimal RNA polymerase II elongation rate. Genes Dev. 2014; 28:2663–2676. 25452276 10.1101/gad.252106.114 PMC4248296

[43] Herzel L. , Straube K., Neugebauer K.M. Long-read sequencing of nascent RNA reveals coupling among RNA processing events. Genome Res. 2018; 28:1008–1019. 29903723 10.1101/gr.232025.117 PMC6028129

[44] Drexler H.L. , Choquet K., Churchman L.S. Splicing kinetics and coordination revealed by direct nascent RNA sequencing through nanopores. Mol. Cell. 2020; 77:985–998.e8. 31839405 10.1016/j.molcel.2019.11.017 PMC7060811

[45] Fong N. , Bentley D.L. Capping, splicing, and 3′ processing are independently stimulated by RNA polymerase. II: different functions for different segments of the CTD. Genes Dev. 2001; 15:1783–1795. 11459828 10.1101/gad.889101 PMC312735

[46] de la Mata M. , Kornblihtt A.R. RNA polymerase II C-terminal domain mediates regulation of alternative splicing by SRp20. Nat. Struct. Mol. Biol. 2006; 13:973–980. 17028590 10.1038/nsmb1155

[47] Dutta S. , Singhal P., Agrawal P., Tomer R., Kritee K., Khurana E., Jayaram B. A physico-chemical model for analyzing DNA sequences. J. Chem. Inf. Model. 2006; 46:78–85. 16426042 10.1021/ci050119x

[48] Singhal P. , Jayaram B., Dixit S.B., Beveridge D.L. Prokaryotic gene finding based on physicochemical characteristics of codons calculated from molecular dynamics simulations. Biophys. J. 2008; 94:4173–4183. 18326660 10.1529/biophysj.107.116392 PMC2480686

[49] Khandelwal G. , Bhyravabhotla J. A phenomenological model for predicting melting temperatures of DNA sequences. PLoS One. 2010; 5:e12433. 20865157 10.1371/journal.pone.0012433 PMC2928768

[50] Khandelwal G. , Jayaram B. DNA-water interactions distinguish messenger RNA genes from transfer RNA genes. J. Am. Chem. Soc. 2012; 134:8814–8816. 22551381 10.1021/ja3020956

[51] Khandelwal G. , Gupta J., Jayaram B. DNA-energetics-based analyses suggest additional genes in prokaryotes. J. Biosci. 2012; 37:433–444. 22750981 10.1007/s12038-012-9221-7

[52] Khandelwal G. , Lee R.A., Jayaram B., Beveridge D.L. A statistical thermodynamic model for investigating the stability of DNA sequences from oligonucleotides to genomes. Biophys. J. 2014; 106:2465–2473. 24896126 10.1016/j.bpj.2014.04.029 PMC4052264

[53] Singh A. , Mishra A., Khosravi A., Khandelwal G., Jayaram B. Physico-chemical fingerprinting of RNA genes. Nucleic Acids Res. 2017; 45:e47. 27932456 10.1093/nar/gkw1236 PMC5397174

[54] Mishra A. , Siwach P., Singhal P., Jayaram B. ChemGenome2.1: an ab initio gene prediction software. Methods Mol. Biol. 2019; 1962:121–138. 31020557 10.1007/978-1-4939-9173-0_7

[55] Mishra A. , Siwach P., Misra P., Jayaram B., Bansal M., Olson W.K., Thayer K.M., Beveridge D.L. Toward a universal structural and energetic model for prokaryotic promoters. Biophys. J. 2018; 115:1180–1189. 30172386 10.1016/j.bpj.2018.08.002 PMC6170797

[56] Bolshoy A. , McNamara P., Harrington R.E., Trifonov E.N. Curved DNA without A-A: experimental estimation of all 16 DNA wedge angles. PNAS. 1991; 88:2312–2316. 2006170 10.1073/pnas.88.6.2312 PMC51221

[57] Bansal M. , Kumar A., Yella V.R. Role of DNA sequence based structural features of promoters in transcription initiation and gene expression. Curr. Opin. Struct. Biol. 2014; 25:77–85. 24503515 10.1016/j.sbi.2014.01.007

[58] Jammalamadaka S.R. , SenGupta A. Topics in Circular Statistic: Section 1.3. 2001; Singapore World Scientific Press.

[59] Eisenberg E. , Levanon E.Y. Human housekeeping genes, revisited. Trends Genet. 2013; 29:569–574. 23810203 10.1016/j.tig.2013.05.010

[60] Russ J. , Futschik M.E. Comparison and consolidation of microarray data sets of human tissue expression. BMC Genomics. 2010; 11:305. 20465848 10.1186/1471-2164-11-305 PMC2885367

[61] Crooks G.E. , Hon G., Chandonia J.M., Brenner S.E. WebLogo: a sequence logo generator. Genome Res. 2004; 14:1188–1190. 15173120 10.1101/gr.849004 PMC419797

[62] Patel A.A. , Steitz J.A. Splicing double: insights from the second spliceosome. Nat. Rev. Mol. Cell Biol. 2003; 4:960–970. 14685174 10.1038/nrm1259

[63] Temiz N.A. , Donohue D.E., Bacolla A., Luke B.T., Collins J.R. The role of methylation in the intrinsic dynamics of B- and Z-DNA. PLoS One. 2012; 7:e35558. 22530050 10.1371/journal.pone.0035558 PMC3328458

[64] Várnai P. , Djuranovic D., Lavery R., Hartmann B. Alpha/gamma transitions in the B-DNA backbone. Nucleic Acids Res. 2002; 30:5398–5406. 12490708 10.1093/nar/gkf680 PMC140057

[65] Matlin A.J. , Moore M.J. Spliceosome assembly and composition. Adv. Exp. Med. Biol. 2007; 623:14–35. 18380338 10.1007/978-0-387-77374-2_2

[66] Staley J.P. , Woolford J.L. Jr Assembly of ribosomes and spliceosomes: complex ribonucleoprotein machines. Curr. Opin. Cell Biol. 2009; 21:109–118. 19167202 10.1016/j.ceb.2009.01.003 PMC2698946

[67] Roca X. , Akerman M., Gaus H., Berdeja A., Bennett C.F., Krainer A.R. Widespread recognition of 5′ splice sites by noncanonical base-pairing to U1 snRNA involving bulged nucleotides. Genes Dev. 2012; 26:1098–1109. 22588721 10.1101/gad.190173.112 PMC3360564

[68] Kumar A. , Bansal M. Unveiling DNA structural features of promoters associated with various types of TSSs in prokaryotic transcriptomes and their role in gene expression. DNA Res. 2017; 24:25–35. 27803028 10.1093/dnares/dsw045 PMC5381344

