# Supplementary material for: Intron exon boundary junctions in human genome have in-built unique structural and energetic signals
Source: Nucleic Acids Res. 2021 Feb 23;49(5):2674–83. doi: 10.1093/nar/gkab098 (PMC7969029; doi:10.1093/nar/gkab098)

Supplementary File 1

Intron-Exon boundary junctions in human genome have in-built unique structural and energetic signals

Akhilesh Mishra[#], Priyanka Siwach[#], Pallavi Misra, Simran Dhiman, Ashutosh Kumar Pandey, Parul Srivastava, B. Jayaram[*]

Contents

Supplementary Tables:

1. Table S1.1: List of ids of crystal structures of B-DNA obtained from NDB……………………………………..(2)
2. Table S1.2: Values for structural and energy parameters for the unique dinucleotide steps…………..………(3)
3. Table S1.3: Parameter wise percentage of sequence exhibiting signal at exon to intron transition……....….(4)
4. Table S1.4: Parameter wise percentage of sequence exhibiting signal at intron to exon transition……....…....(5)

Supplementary Methodology:

1. Methodology S1.1: Calculation of area enclosed between two vectors and process of optimization of threshold
   - S1.1 (a): Calculation of area enclosed between two vectors…………………………………………….... (6)
   - S1.1 (b): Optimization of threshold………………………………………………………………………... (6)

Supplementary Figures:

1. Figure S1.1: Structural and energy profiles of 401 nucleotides long sequences (Exon to Intron)
   - Figure S1.1 (a): Exon to Intron - Backbone parameters…………………………………………………….. (7)
   - Figure S1.1 (b): Exon to Intron - Inter Base pair parameters………………………………………………. (8)
   - Figure S1.1 (c): Exon to Intron - Intra base pair parameters………………………………………………. (9)
   - Figure S1.1 (d): Exon to Intron - Base Pair Axis parameters……………………………………………… (10)
   - Figure S1.1 (e): Exon to Intron - Energy parameters……………………………………………………….. (10)
2. Figure S1.2: Structural and energy profiles of 401 nucleotides long sequences (Intron to Exon)
   - Figure S1.2 (a): Intron to Exon - Backbone parameters……………………………………………………. (11)
   - Figure S1.2 (b): Intron to Exon - Inter Base pair parameters……………………………………..……. (12)
   - Figure S1.2 (c): Intron to Exon - Intra base pair parameters……………………………………………. (13)
   - Figure S1.2 (d): Intron to Exon - Base Pair Axis parameters……………………………………………… (14)
   - Figure S1.2 (e): Intron to Exon - Energy parameters……………………………………………………...….. (14)
3. Figure S1.3: Optimization of threshold value at μ, μ-σ and at μ-2σ for intron to exon transition……………. (15)
4. Figure S1.4: Optimization of threshold value at μ, μ-σ and at μ-2σ for exon to intron transition…………… (16)
5. Figure S1.5: Heat plots for Dataset I and Dataset II……………………………………………………………….(17)

Table S1.1: List of ids of crystal structures of B-DNA only (without any protein/ligand) obtained from NDB

Sr No.	NDB ids	Sr No.	NDB ids
1.	111d.pdb	38.	250d.pdb
2.	112d.pdb	39.	251d.pdb
3.	113d.pdb	40.	252d.pdb
4.	122d.pdb	41.	272d.pdb
5.	123d.pdb	42.	287d.pdb
6.	126d.pdb	43.	2af1.pdb
7.	167d.pdb	44.	2bna.pdb
8.	194d.pdb	45.	2g1z.pdb
9.	1bna.pdb	46.	2got.pdb
10.	1cgc.pdb	47.	2org.pdb
11.	1d28.pdb	48.	2qef.pdb
12.	1d29.pdb	49.	2qeg.pdb
13.	1d31.pdb	50.	307d.pdb
14.	1d65.pdb	51.	309d.pdb
15.	1d89.pdb	52.	329d.pdb
16.	1d98.pdb	53.	330d.pdb
17.	1d99.pdb	54.	3ajj.pdb
18.	1dcv.pdb	55.	3bna.pdb
19.	1dn9.pdb	56.	3gbi.pdb
20.	1dnm.pdb	57.	3l1q.pdb
21.	1ehv.pdb	58.	3n4n.pdb
22.	1hq7.pdb	59.	3nao.pdb
23.	1ilc.pdb	60.	3q5c.pdb
24.	1n4e.pdb	61.	3ubi.pdb
25.	1ndn.pdb	62.	424d.pdb
26.	1vtd.pdb	63.	425d.pdb
27.	1vte.pdb	64.	458d.pdb
28.	1wqy.pdb	65.	4b8d.pdb
29.	1wqz.pdb	66.	4bna.pdb
30.	1zf5.pdb	67.	4glc.pdb
31.	1zfc.pdb	68.	4glg.pdb
32.	1zfe.pdb	69.	4gs2.pdb
33.	1zff.pdb	70.	4gsg.pdb
34.	1zfg.pdb	71.	4gsi.pdb
35.	1zfh.pdb	72.	4j2i.pdb
36.	1zfm.pdb	73.	7bna.pdb
37.	237d.pdb	74.	9bna.pdb

Table S1.2: Values for structural and energy parameters for the unique dinucleotide steps

	AA/TT	AT	AC/GT	AG/CT	TA	CG	CC/GG	GA/TC	CA/TG	GC
**X Disp.**	-0.246	-0.178	-0.031	0.486	0.102	0.705	0.585	-0.158	0.312	0.593
**Y Disp.**	0.004	-0.026	-0.051	-0.029	-0.286	0.060	-0.078	0.080	-0.068	0.083
**Inclination**	-0.614	-0.260	0.680	1.825	0.547	2.911	9.909	0.526	3.005	0.365
**Tip**	0.597	0.461	2.510	3.723	-0.766	0.303	3.128	0.752	0.175	2.507
**Ax-Bend**	1.422	1.213	1.250	1.652	1.471	1.489	2.586	1.178	1.055	1.244
**Shear**	0.128	0.014	0.055	-0.064	0.026	0.355	1.224	0.050	-0.002	0.021
**Stretch**	-0.180	-0.134	-0.125	-0.168	-0.177	-0.358	-1.123	-0.199	-0.149	-0.137
**Stagger**	0.121	0.147	0.573	-0.146	0.085	0.561	1.670	0.215	0.156	0.284
**Buckle**	-1.482	0.279	-5.579	-0.850	-0.326	2.413	8.842	-1.169	0.076	-0.983
**Propel**	-15.308	-16.536	-9.838	-11.734	-14.037	-3.280	5.707	-13.135	-10.824	-10.443
**Opening**	3.159	4.110	2.110	-3.668	3.305	-0.849	5.719	0.943	2.073	-1.116
**Shift**	-0.110	-0.060	-0.120	-0.270	-0.050	0.240	0.380	0.040	-0.070	-0.280
**Slide**	-0.200	-0.440	0.730	0.210	0.370	0.680	0.710	-0.200	0.520	0.170
**Rise**	3.250	3.310	3.660	3.020	3.390	3.330	2.960	3.320	3.290	3.350
**Tilt**	0.630	-0.900	-4.750	3.690	-2.710	1.750	-1.620	2.210	-3.270	-2.680
**Roll**	-0.080	-2.620	-0.600	-7.270	1.740	4.290	-2.550	2.690	1.540	-5.280
**Twist**	35.670	33.420	34.790	31.000	32.050	37.380	29.490	37.740	36.740	31.090
**H-Rise**	3.270	3.330	2.970	3.060	3.410	3.330	2.730	3.330	3.210	3.290
**H-Twist**	35.560	33.680	37.600	19.060	32.000	39.100	31.570	37.810	37.250	30.560
**Alpha**	-55.550	-51.913	-45.474	-36.214	-50.820	-43.178	-38.322	-42.611	-52.023	-48.267
**Beta**	51.044	59.981	42.118	36.155	29.230	57.555	39.807	54.835	41.346	36.968
**Gamma**	50.772	47.175	51.861	43.870	53.740	51.502	44.997	53.374	35.389	36.646
**Delta**	128.408	125.847	126.878	129.955	127.846	132.616	129.998	131.432	131.452	129.951
**Epsilon**	-5.924	-31.230	-34.321	-43.587	-37.772	-67.013	-56.716	-8.081	-75.781	-70.502
**Zeta**	-96.656	-93.609	-55.767	-34.570	-61.970	-62.643	-61.650	-94.004	-60.380	-63.978
**Chi**	-110.254	-113.738	-116.547	-88.341	-106.740	-106.586	-103.913	-110.983	-91.503	-109.979
**Phase**	118.784	107.855	106.334	73.080	119.920	103.300	96.172	118.188	73.933	84.877
**Amplitude**	38.996	40.016	38.650	38.941	37.824	37.951	37.361	37.945	38.744	38.594
**Hydrogen Bonding Energy**	-5.440	-5.350	-7.140	-6.270	-5.830	-8.050	-8.480	-7.800	-7.010	-8.720
**Stacking Energy**	-26.710	-27.200	-27.730	-26.890	-26.900	-27.930	-26.280	-26.780	-27.150	-28.130
**Solvation Energy**	-171.840	-173.700	-171.110	-174.930	-174.350	-176.880	-166.760	-167.600	-179.010	-165.580

Table S1.3: Parameter wise percentage of sequence exhibiting signal at exon to intron transition.

Parameter Name	Min	Max	Mean	Std.	Below Threshold	Above Threshold	Total	Percentage
X Disp.	2.36	46.18	15.72	5.96	5941	322427	328368	98.19
Y Disp.	2.19	47.87	14.85	5.17	1672	326696	328368	99.49
Inclination	1.57	48.15	15.66	5.86	5525	322843	328368	98.32
Tip	2.59	51.72	15.28	5.63	3613	324755	328368	98.90
Ax-Bend	2.13	48.03	15.00	5.48	4378	323990	328368	98.67
Shear	1.21	46.20	15.55	5.80	5998	322370	328368	98.17
Stretch	0.57	48.02	16.03	6.11	7643	320725	328368	97.67
Stagger	1.88	46.55	15.46	5.74	5257	323111	328368	98.40
Buckle	1.81	46.78	15.14	5.59	4881	323487	328368	98.51
Propel	1.90	47.71	15.73	5.91	5790	322578	328368	98.24
Opening	3.11	42.84	14.66	5.08	1321	327047	328368	99.60
Shift	1.74	44.20	14.65	5.31	3807	324561	328368	98.84
Slide	2.24	48.55	15.36	5.64	3736	324632	328368	98.86
Rise	2.27	48.82	14.63	5.20	2218	326150	328368	99.32
Tilt	2.28	45.09	14.56	5.08	1636	326732	328368	99.50
Roll	3.13	40.25	14.39	4.91	733	327635	328368	99.78
Twist	2.65	47.92	14.80	5.24	1889	326479	328368	99.42
H-Rise	1.65	48.79	15.21	5.56	3623	324745	328368	98.90
H-Twist	2.82	45.89	14.53	4.93	819	327549	328368	99.75
Alpha	2.12	46.94	15.43	5.79	5420	322948	328368	98.35
Beta	2.64	43.71	14.85	5.18	1562	326806	328368	99.52
Gamma	2.23	45.67	15.13	5.41	2437	325931	328368	99.26
Delta	2.11	53.30	15.37	5.73	4778	323590	328368	98.54
Epsilon	1.98	46.00	15.45	5.75	4589	323779	328368	98.60
Zeta	1.78	49.35	15.22	5.63	4463	323905	328368	98.64
Chi	2.74	49.47	14.58	5.00	735	327633	328368	99.78
Phase	2.13	47.29	15.11	5.54	3482	324886	328368	98.94
Amplitude	2.25	49.19	15.13	5.52	3174	325194	328368	99.03
Hydrogen Bond Energy	1.84	51.92	15.72	6.04	6924	321444	328368	97.89
Stacking Energy	2.50	44.40	14.50	5.10	1832	326536	328368	99.44
Solvation Energy	2.54	44.01	14.07	4.85	875	327493	328368	99.73

Table S1.4: Parameter wise percentage of sequence exhibiting signal at intron to exon transition.

Parameter Name	Min	Max	Mean	Std.	Below Threshold	Above Threshold	Total	Percentage
X Disp.	0.66	47.34	15.83	6.00	5781	322587	328368	98.24
Y Disp.	1.86	44.27	14.68	5.09	1625	326743	328368	99.51
Inclination	1.25	45.02	15.79	5.89	5187	323181	328368	98.42
Tip	0.31	48.59	15.28	5.67	3833	324535	328368	98.83
Ax-Bend	1.03	43.97	15.08	5.47	3777	324591	328368	98.85
Shear	1.43	44.44	15.69	5.81	5448	322920	328368	98.34
Stretch	0.43	46.65	16.18	6.13	7014	321354	328368	97.86
Stagger	1.39	45.60	15.62	5.77	4951	323417	328368	98.49
Buckle	0.60	43.90	15.25	5.59	4378	323990	328368	98.67
Propel	1.11	46.74	15.87	5.95	5450	322918	328368	98.34
Opening	2.35	44.85	14.63	5.05	1215	327153	328368	99.63
Shift	2.05	44.56	14.78	5.34	3414	324954	328368	98.96
Slide	1.41	45.19	15.50	5.72	4049	324319	328368	98.77
Rise	0.72	46.99	14.64	5.18	2070	326298	328368	99.37
Tilt	1.47	47.79	14.60	5.09	1653	326715	328368	99.50
Roll	1.48	41.71	14.45	4.93	731	327637	328368	99.78
Twist	0.40	45.64	14.88	5.26	1803	326565	328368	99.45
H-Rise	0.87	45.89	15.29	5.61	3704	324664	328368	98.87
H-Twist	1.60	45.38	14.54	4.93	762	327606	328368	99.77
Alpha	0.15	45.97	15.41	5.80	5503	322865	328368	98.32
Beta	0.66	42.63	14.91	5.22	1602	326766	328368	99.51
Gamma	1.38	44.70	15.21	5.43	2426	325942	328368	99.26
Delta	0.39	49.34	15.24	5.71	5005	323363	328368	98.48
Epsilon	0.38	48.21	15.59	5.79	4663	323705	328368	98.58
Zeta	0.84	45.48	15.28	5.69	4887	323481	328368	98.51
Chi	1.13	47.92	14.56	5.01	780	327588	328368	99.76
Phase	1.62	44.97	15.14	5.55	3562	324806	328368	98.92
Amplitude	0.39	47.00	15.19	5.54	3119	325249	328368	99.05
Hydrogen Bond Energy	0.30	48.33	15.79	6.07	6970	321398	328368	97.88
Stacking Energy	0.51	46.85	14.52	5.11	1894	326474	328368	99.42
Solvation Energy	0.53	46.49	14.08	4.83	803	327565	328368	99.76

Methodology S1.1: Calculation of area enclosed between two vectors and process of optimization of threshold.

S1.1 (a): Calculation of area enclosed between two vectors:

Let $f(x)$and $g(x)$ be the function of exon-intron junction and CDS vectors respectively. Then the area between these two curves is given by:

$$\mathbf{A} = \int_{\boldsymbol{a}}^{\boldsymbol{b}} (|\boldsymbol{f}(\boldsymbol{x}) - \boldsymbol{g}(\boldsymbol{x})|)$$

Here a and b are respectively the first and last values of both vectors. By using the trapezoidal approximation method the area can be approximated without the need for exact function equations. The area calculation for both the curves of function $f(x)$ and $g(x)$ then reduces to:

$$A_{f(x)} = \sum_{\lambda=1}^{n} \frac{f(x_{\lambda-1})+f(x_{\lambda})}{2} \Delta x_{\lambda}$$

$$A_{g(x)} = \sum_{\lambda=1}^{n} \frac{g(x_{\lambda-1})+g(x_{\lambda})}{2} \Delta x_{\lambda}$$

Where n is the number of sections of vectors.

S1.1 (b): Optimization of threshold:

To decide the optimal threshold to separate junction and CDS vector, the enclosed area for every pair within parameters were selected and its mean (μ) and standard deviation (σ) were calculated. Then we chose 3 different threshold values for area namely μ, μ-σ, μ-2σ to separate vectors within each parameter into two groups of above and below threshold. Using the two conditions namely 'area enclosed between the individual junction and CDS vectors', and 'morphology of both the vectors', were used to decide the optimal threshold. Supplementary figure S3 (a-b) shows that at the threshold μ-2σ, the mean curve of all the parameters for junction and CDS vectors have very low enclosed area and morphology becomes similar. This is not observed at threshold values above this criteria.

At μ – 3*σ the threshold (θ) will become 0 as it was observed that for every parameter, σ is approx. one third of μ.

Figure S1.1: Structural and energy profiles of 401 nucleotides long sequences: Exon-Intron boundary sequences (from exon to intron), with 0 being the last nucleotide of exon (green line) and coding sequences (red line).

Figure S1.1 (a): Exon to Intron - Backbone parameters

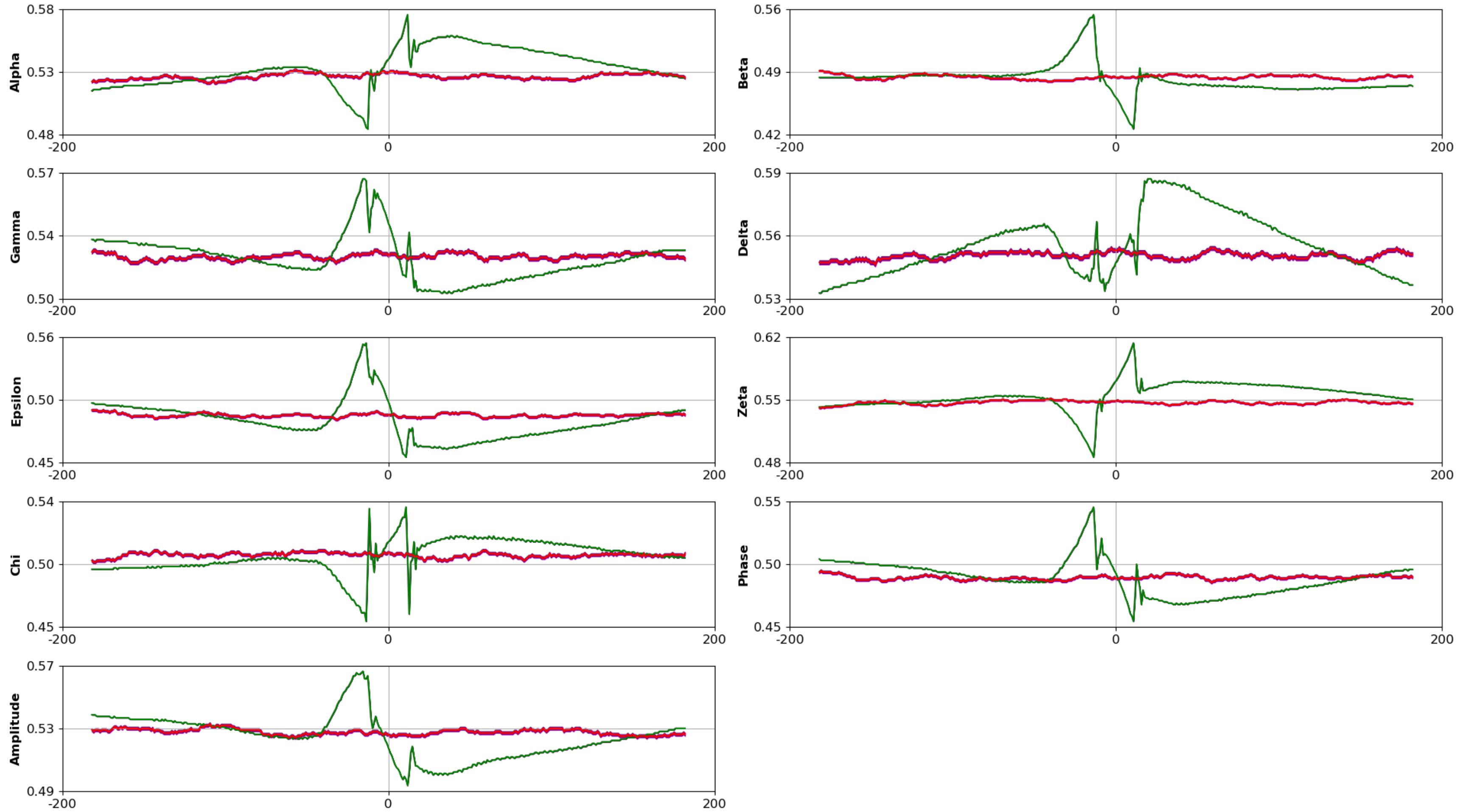

Figure S1.1 (b): Exon to Intron - Inter Base pair parameters

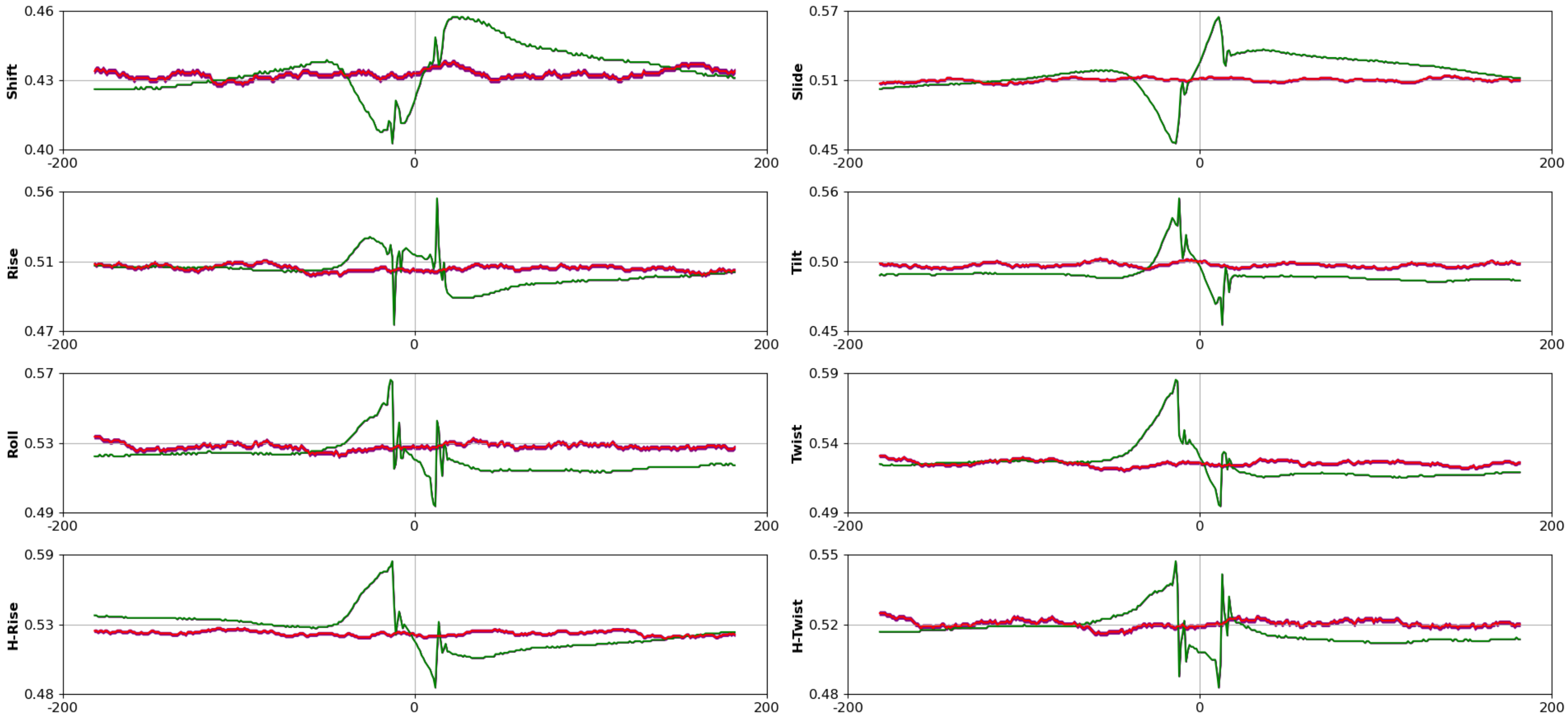

Figure S1.1 (c): Exon to Intron - Intra base pair parameters

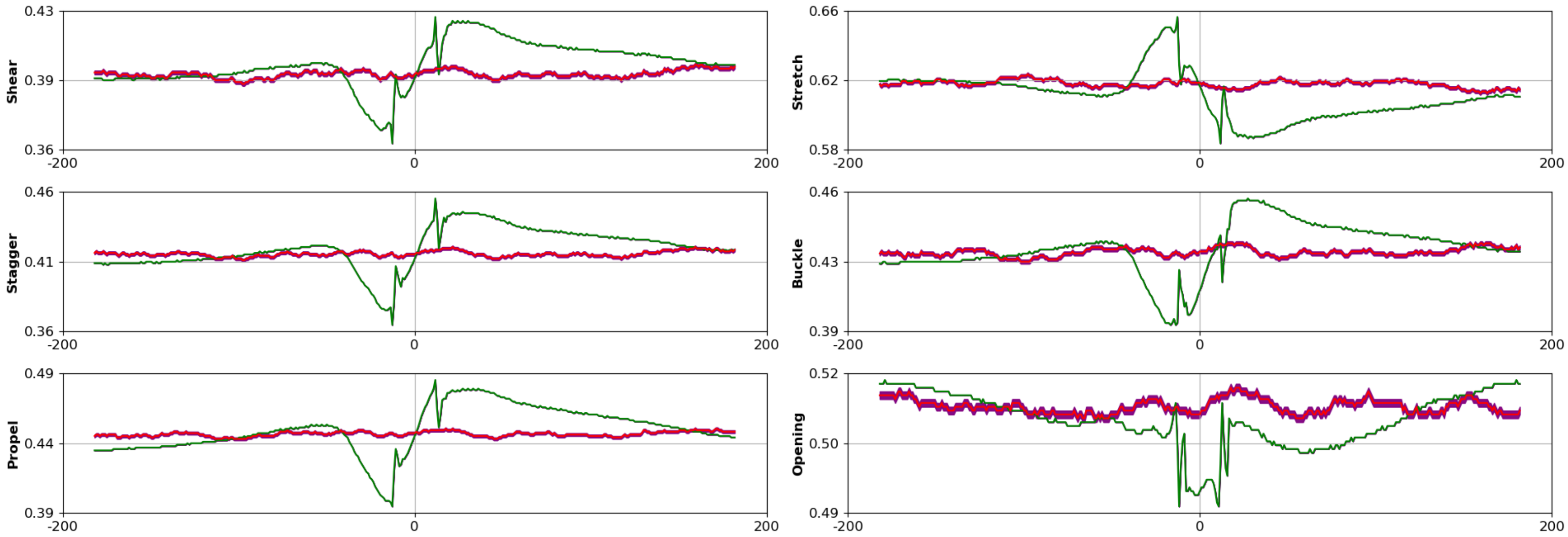

Figure S1.1 (d): Exon to Intron – Base Pair Axis parameters

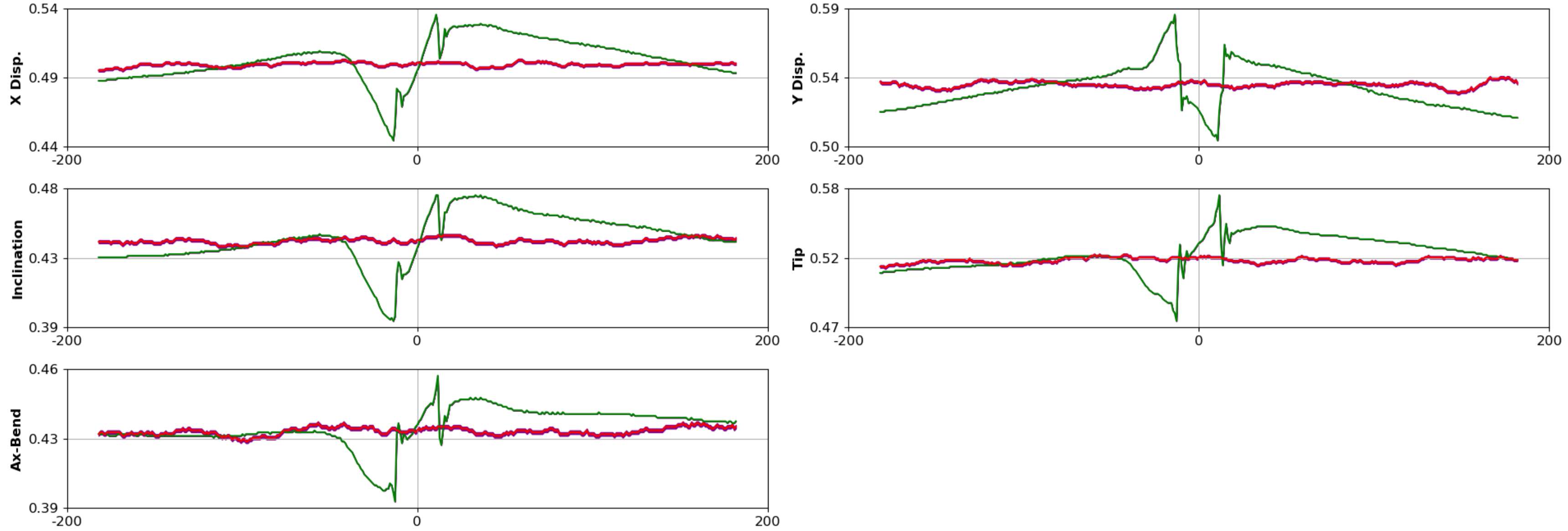

Figure S1.1 (e): Exon to Intron - Energy parameters

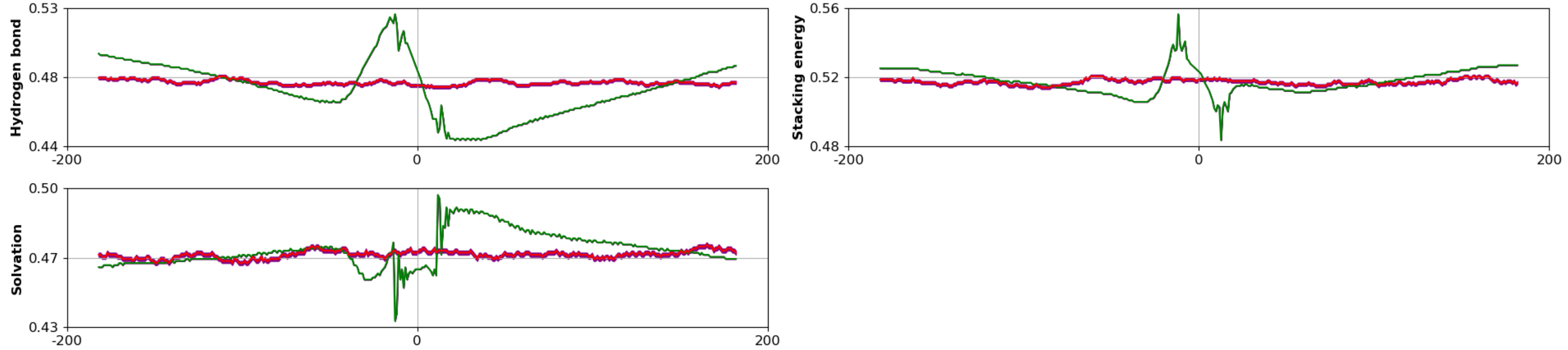

Figure S1.2: Structural and energy profiles of 401 nucleotides long sequences: Intron-Exon boundary sequences (from intron to exon), with 0 being the first nucleotide of exon (green line) and coding sequences (red line).

Figure S1.2 (a): Intron to Exon - Backbone parameters

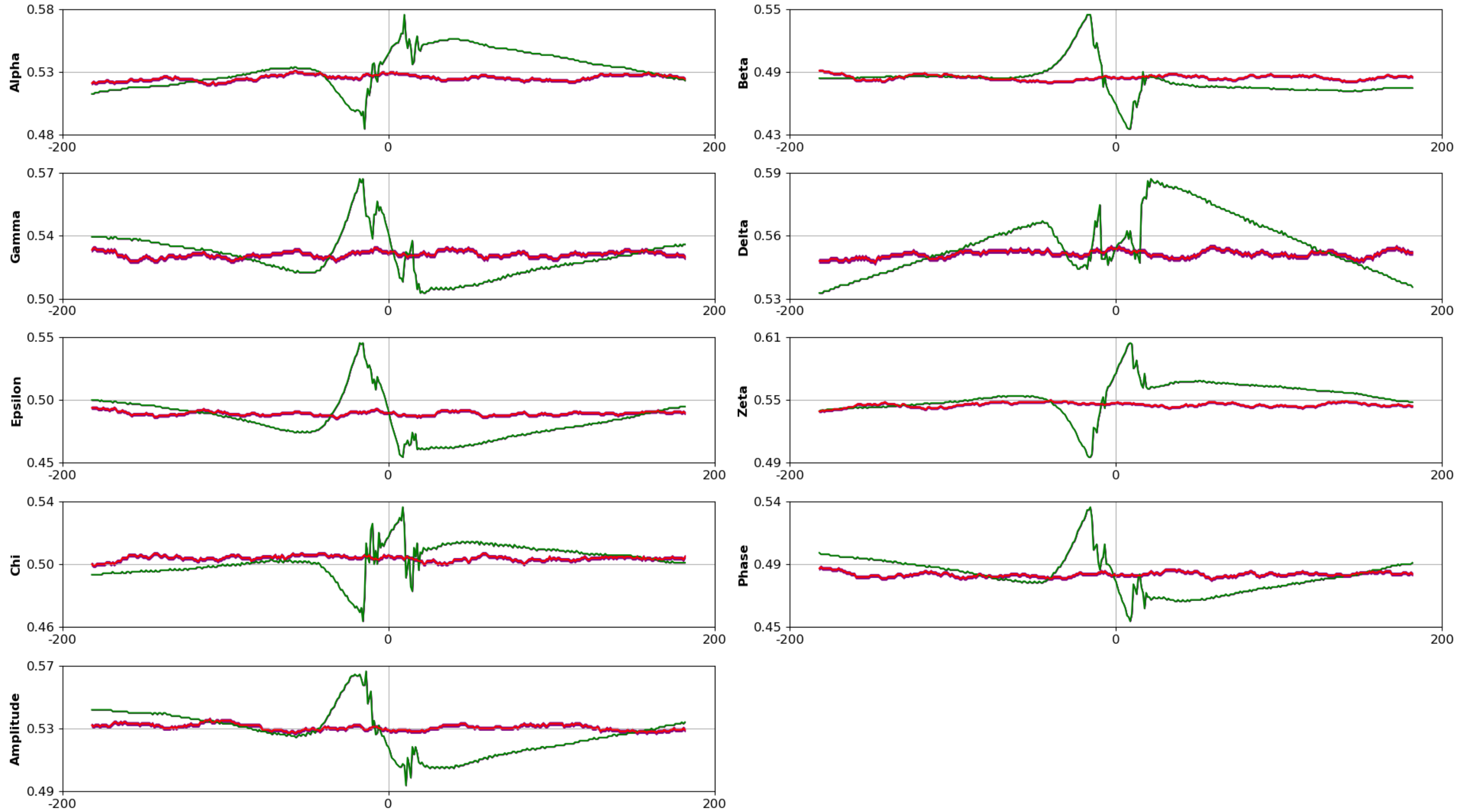

Figure S1.2 (b): Intron to Exon - Inter base pair parameters

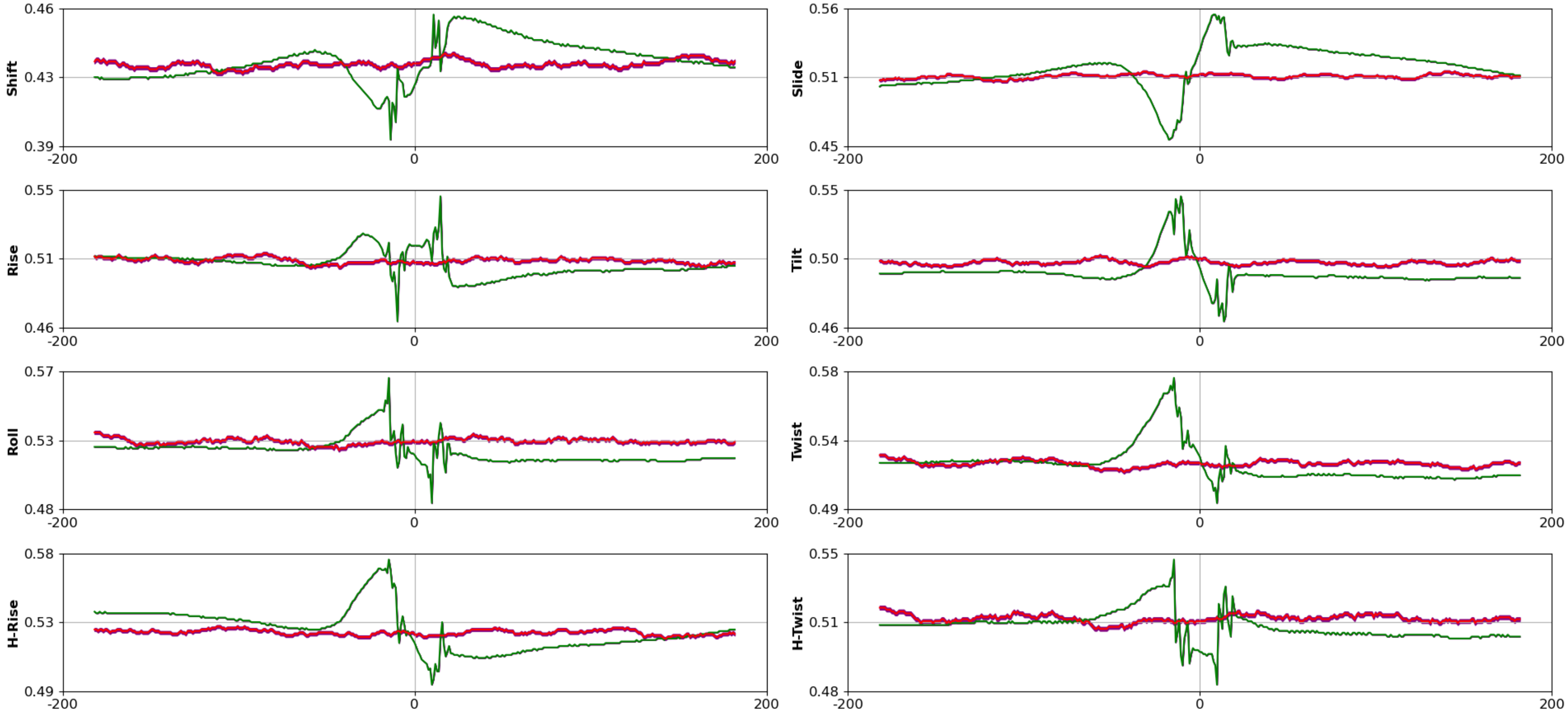

Figure S1.2 (c): Intron to Exon - Intra base pair parameters

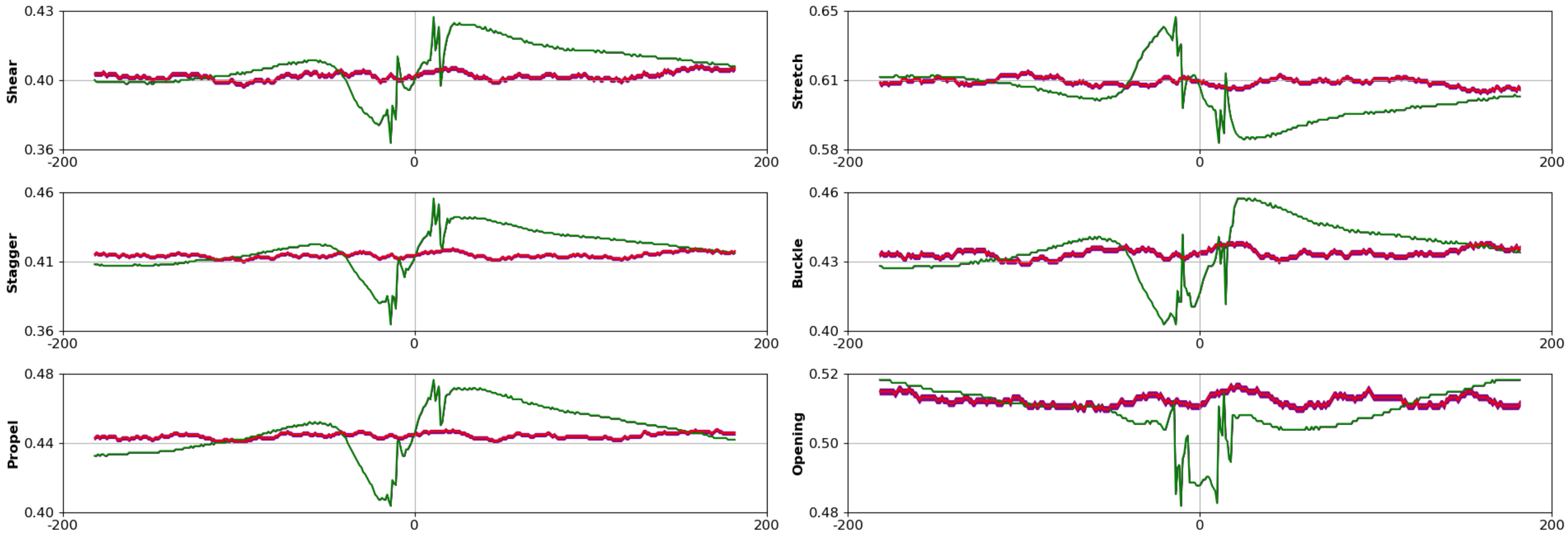

Figure S1.2 (d): Intron to Exon – Base Pair Axis parameters

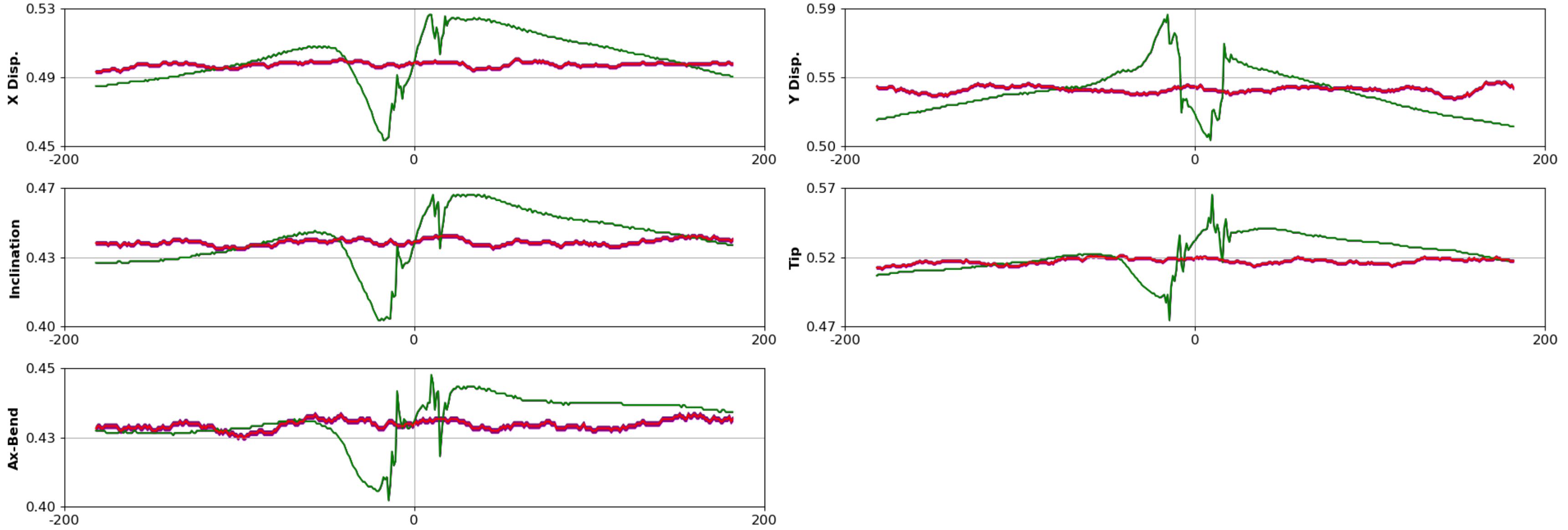

Figure S1.2 (e): Intron to Exon - Energy parameter

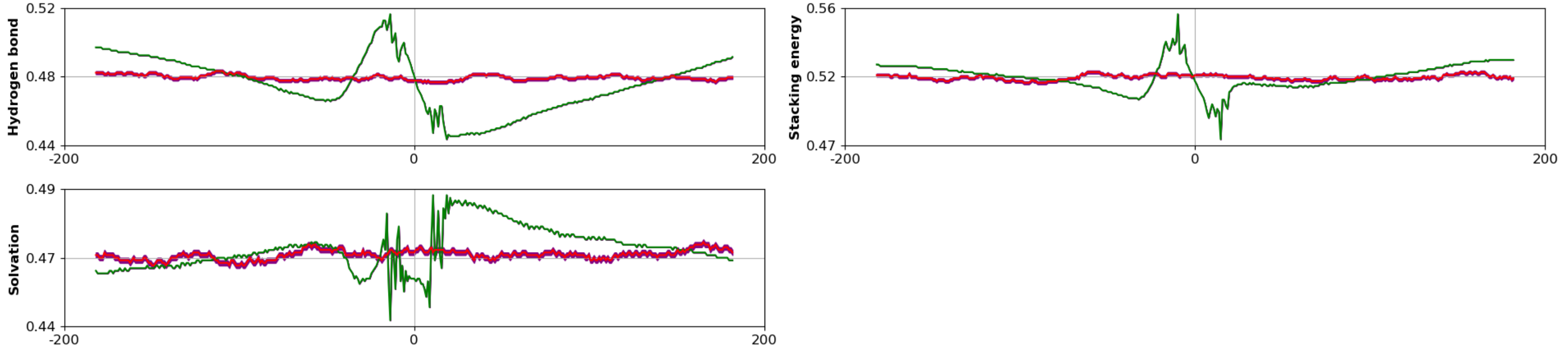

Figure S1.3: Optimization of threshold value at μ, μ-σ and at μ-2σ for intron to exon transition. The intron to exon transition vector is represented in blue color while the CDS vector is represented in Orange color.

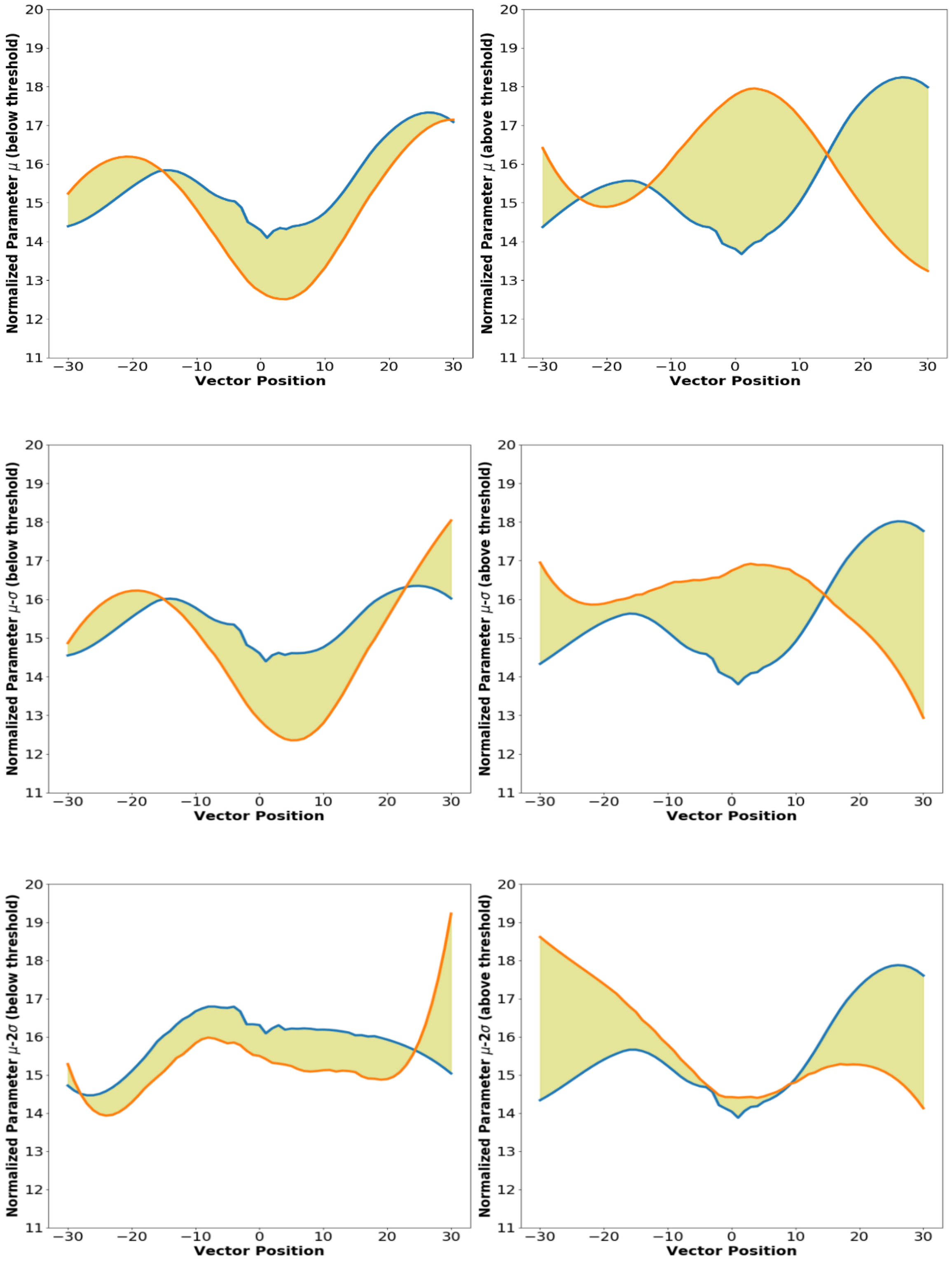

Figure S1.4: Optimization of threshold value at μ, μ-σ and at μ-2σ for exon to intron transition. The exon to intron transition vector is represented in blue color while the CDS vector is represented in Orange color.

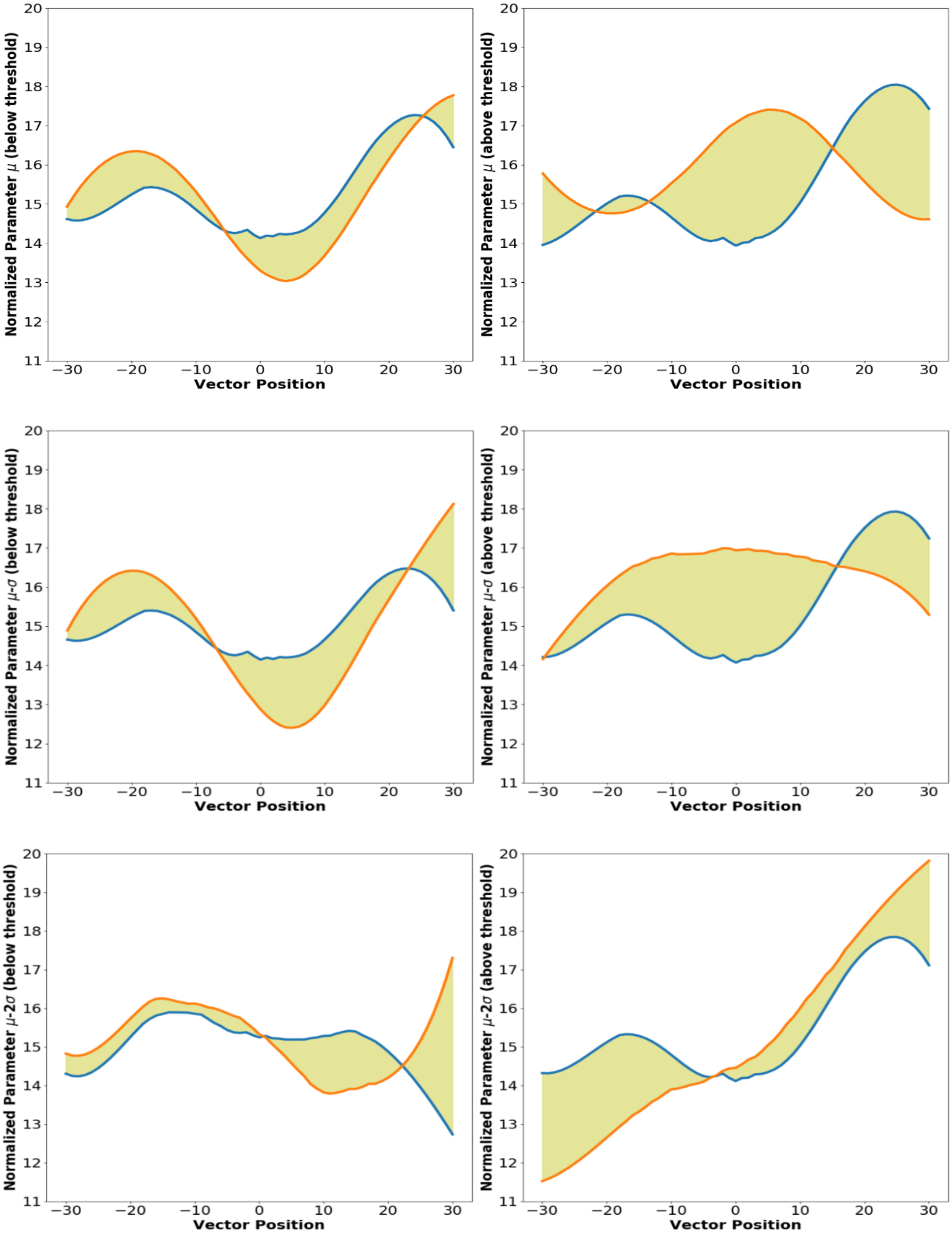

Figure S1.5: Heat plots of Dataset I and Dataset II generated using position specific correlation coefficients values between all pairs of twenty-six parameters [X Disp. (A), Inclination (B), Tip (C), Ax-Bend (D), Shear (E), Stretch (F), Stagger (G), Buckle (H), Propel (I), Shift (J), Slide (K), Rise (L), Tilt (M), Roll (N), Twist (O), H-Rise (P), H-Twist (Q), Beta (R) Gamma (S), Delta (T), Epsilon (U), Zeta (V), Chi (W), Phase (X), Amplitude (Y), Hydrogen Bond Energy (Z)].

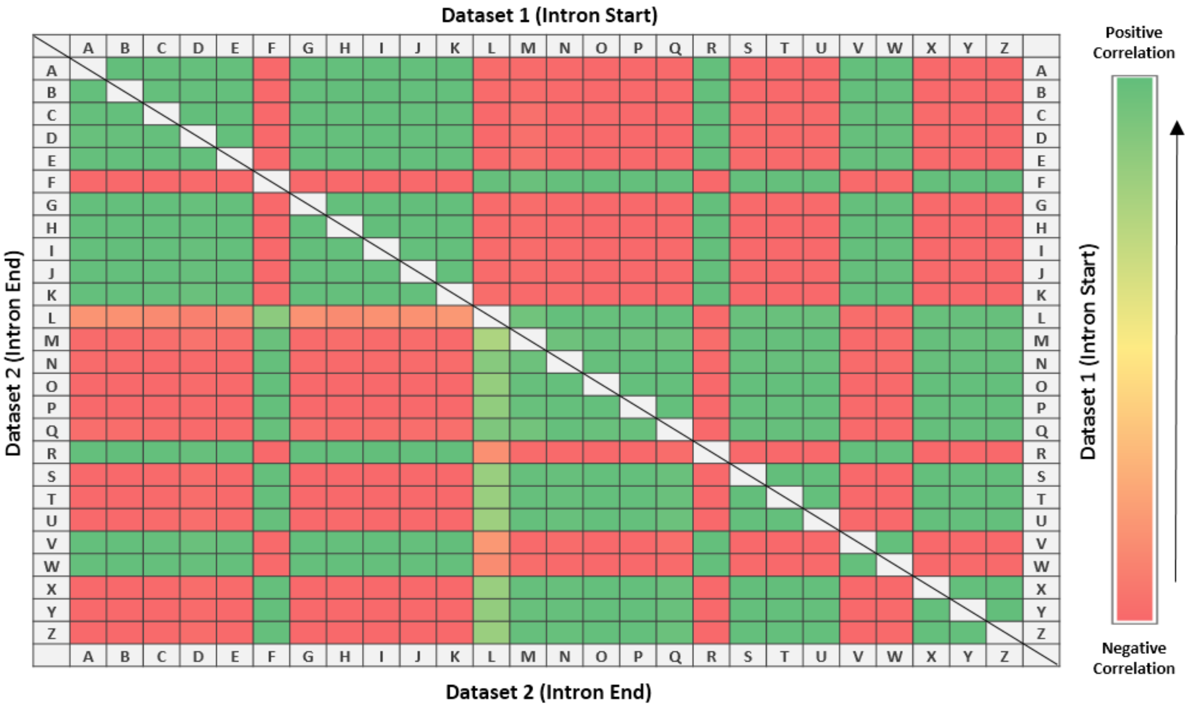

Supplement: gkab098_Supplemental_Files [file gkab098_supplemental_files.zip › Supplementary_File_S1_R2.pdf]
